# CLIMBS: Assessing Carbohydrate–Protein Interactions through a Graph Neural Network Classifier Using Synthetic Negative Data

**DOI:** 10.1021/acs.jcim.6c00126

**Published:** 2026-04-03

**Authors:** Yijie Luo, Fabio Parmeggiani

**Affiliations:** † School of Biochemistry, 1980 University of Bristol, University Walk, Bristol BS8 1TD, U.K.; ‡ School of Chemistry, 1980 University of Bristol, Cantock’s Close, Bristol BS8 1TS, U.K.; § School of Pharmacy and Pharmaceutical Sciences, 2112 Cardiff University, Redwood Building, King Edward VII Ave, Cardiff CF10 3NB, U.K.

## Abstract

Carbohydrate–protein interactions are essential for biological processes, such as cellular signaling and metabolism, and represent a large pool of untapped targets for diagnostics and therapeutics. However, current design and prediction methods fail to accurately evaluate the affinity and specificity of proteins for carbohydrates such as glucose and galactose. Here, we describe a machine learning classifier, named CLIMBS, as a novel evaluation method for protein–carbohydrate interactions and train it on crystal structures and synthetic data from unsuccessfully designed structures to effectively assess whether carbohydrate–protein complexes represent realistic, native-like structures. Compared to other methods, CLIMBS has outstanding accuracy and excellent carbohydrate specificity, supported by high AUROC and MCC values, subsecond runtime per sample, minimal bias toward either negative or positive samples, and can be employed to improve the selection of successful docking and design models of carbohydrate–protein complexes.

## Graphical abstract

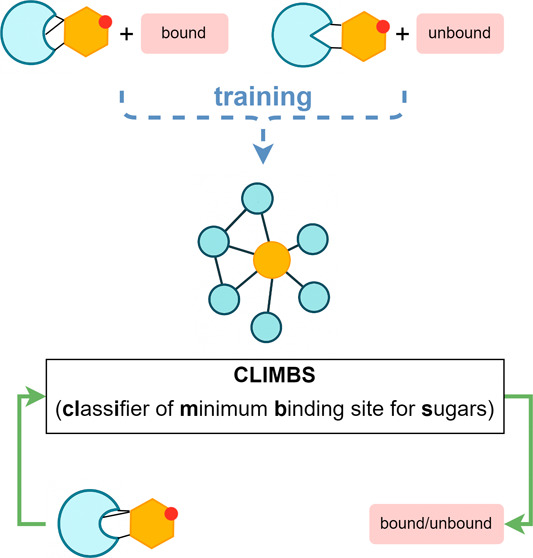

## Introduction

Carbohydrate recognition by proteins is a fundamental step in a number of biological processes, from glycolysis^1^ to assembly of specific glycosylation patterns and formation of polysaccharide materials,^2^ either for energy storage, as for starch,^3^ or for structural use, such as cellulose.^4^ In terms of information and communication, large groups of lectins act as receptors for glycans and polysaccharides and play key roles in cellular signaling and adhesion.^5^ Identification of receptors and binding partners and specificity predictions have become fundamental in understanding interactions between the glycome and the proteome. Although high-throughput^6^ and glycan specificity computational models have been developed,^7,8^ most of the molecular details for specific interactions remain routinely out of experimental grasp, and are addressed through docking and modeling of small molecule–protein interactions.^9,10^

Despite being well-developed, physics-based evaluation methods still face significant challenges in accurately assessing protein–carbohydrate interactions. Generalized scoring functions applied to protein–protein or protein–small molecule complexes have poor performance on protein–carbohydrate complexes, for example, AMBER,^11^ Rosetta,^12^ and AutoDock4.^13^

With the rapid advancement of artificial intelligence in the past decade, knowledge-based approaches have increasingly been employed to evaluate protein–small molecule interactions but have received less focus on carbohydrate. GNINA^14^ and Sfcnn^15^ use convolutional neural networks (CNN) and present complex structures in 3D-grid voxels. CSM-lig^16^ and DeepDOCK^17^ use graph neural networks (GNN), presenting complex structures as graphs with additional physicochemical characteristics as features. Generative models such as DIFFDOCK^18^ and AlphaFold3 ^19^ have intrinsic confidence models to rank and assess the quality of the generated structures.

Some approaches introduce tailored biases to capture the features of protein–carbohydrate interactions appropriately. For example, the GLYCAM06 energy function^20^ and the CSM-carbohydrate model^21^ introduce additional sugar-related energy terms to enhance the fitness of structures containing carbohydrates. However, when considering the whole complex or interface, they can hardly capture weak, rare, but essential interactions for sugar binding, such as the CH–π interaction.

The development of machine learning methods can be extended to protein–carbohydrate interactions, although this remains challenging. Compared to standalone protein structures, there is no available large data set for protein–carbohydrate complexes on which unsupervised learning methods can rely. When looking at available structural information, among 211,432 protein structures in the protein data bank (PDB),^22^ only 12.33% (26,705 structures) of them contained carbohydrates as of February 2024. And if we consider redundancy, structures with resolution lower than 3 Å, structures where the carbohydrate participates in the binding interface, the number drops to 3.84% (8119 structures). In contrast, supervised learning methods, such as classifiers, require much smaller data sets to achieve reasonable performances,^23^ although appropriately labeled. While selected protein–carbohydrate complexes in the PDB can provide positive samples (“binding”), negative samples (“not-binding”) for complexes, where the carbohydrate is positioned unfavorably in a binding pocket, can only be computationally generated. Interestingly, reasonable nonbinders can be easily generated, since even state-of-the-art protein design methods have achieved success rates of only 0.67%^24^ for small molecule binders. This implies that a large synthetic data set of designed binders for target carbohydrates could provide realistic and challenging negative samples for training supervised learning methods.

Based on the features of carbohydrate–protein complex data, we have developed a graph neural network (GNN)-based model: CLIMBS, a **cl**ass**i**fier of **minimal b**inding sites for **s**ugars. We trained it using positive experimental data and synthetic negative data and then compared its performance in discriminating native and computationally generated complexes with those of established scoring methods. We then explored the potential for retraining on new types of sugars and discriminating complexes with untrained sugars. Furthermore, CLIMBS was used to improve the assessment of native-like interface in docking^25^ and design.^26^

## Results

### Overview of CLIMBS

CLIMBS was developed as a graph neural network classifier to validate atomistic models of carbohydrate–protein complexes. CLIMBS takes carbohydrate–protein complex structures as inputs, and it outputs 1 or 0 indicating predictions as “binding” or “nonbinding”. The binding interface, rather than the whole complex, is used as the model input to focus on binding site geometry and reduce the runtime. Atom-level geometric information is used in the form of graphs to capture the details of interactions. Due to the structural similarity between carbohydrates, fragment-level information (including groups of neighboring atoms, such as hydroxyl groups or carbon rings, see Figure 1D,E) is processed within a pooling layer, facilitating learning across various types of sugars with shared features.^27,28^

**Figure 1:**
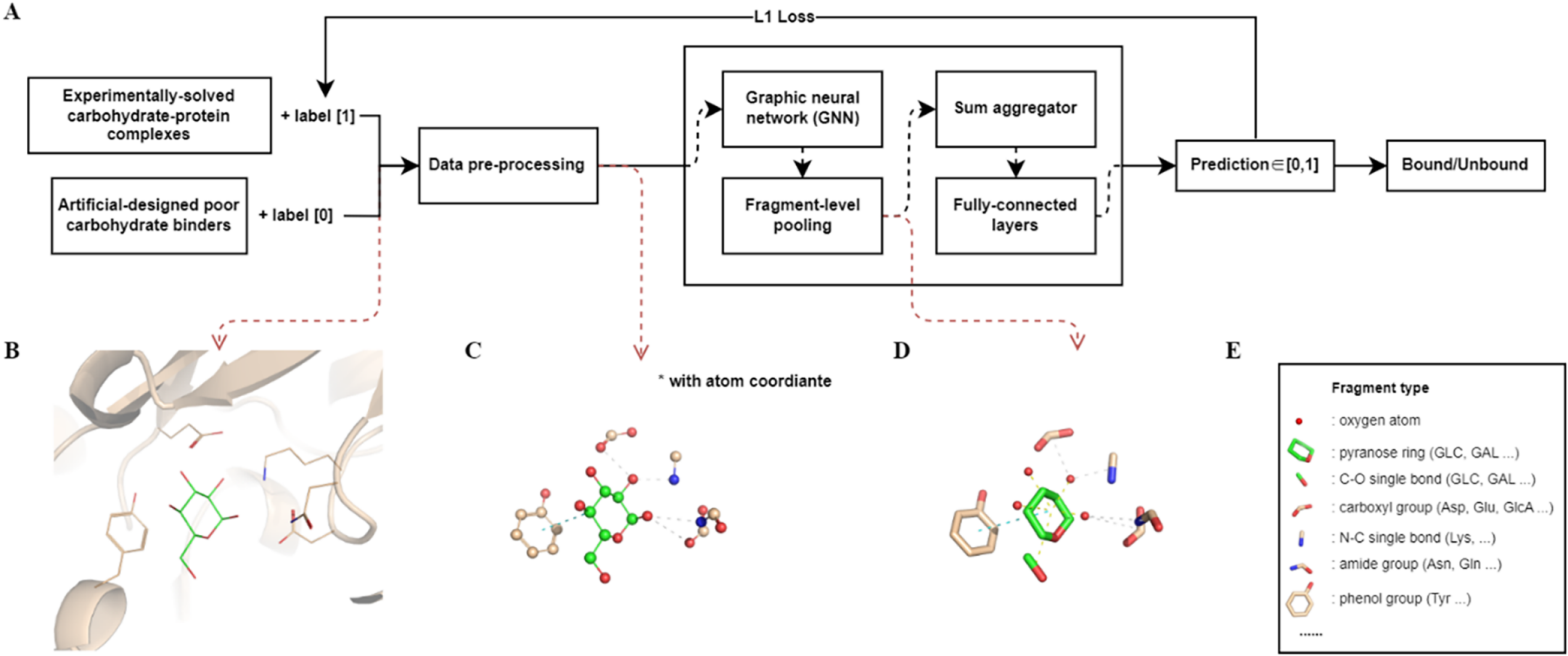
Flowchart of CLIMBS. (A) Overview of CLIMBS. (B) PDB file as input structure data of the model. (C) Structure data becomes an atom-level graph with atom coordinates for each node after data preprocessing. (D) In CLIMBS, structural data becomes a fragment-level graph after a pooling layer. (E) Legend of fragment types shown. GLC: glucose, GAL: galactose, GlcA: glucuronic acid.

A structure library^29^ was built to include 19 different carbohydrates from experimentally determined carbohydrate–protein complexes that were used as positive samples, while suboptimal computationally designed carbohydrate–protein complexes for the same carbohydrates were introduced as negative samples (see Methods). The library (see Supplementary Table 1) mirrors the Protein Data Bank, with seven monosaccharide types (glucose, glucosamine, galactose, L-fucose, mannose, sialic acid, and fructose) accounting for 79% of all complexes.

CLIMBS predicts binding between carbohydrates and proteins with an overall accuracy of 88.90%. The model was trained on 2438 positive and 2429 negative samples (*db_w1*, see Supplementary Data 3a). The binding predictions of different types of sugar–protein complexes were tested on a separate set of complexes containing either carbohydrates used during training or unseen sugars (Figure 2). A minimal accuracy of 70.00% was observed for disaccharides constructed by N-acetyl-β-d-galactosamine and β-d-glucuronic acid (not used in training), and a maximal accuracy of 96.13% for β-D-fructose. Carbohydrates less represented in the library, such as ribose and arabinose, still reach accuracies similar to those of the most abundant ones.

**Figure 2:**
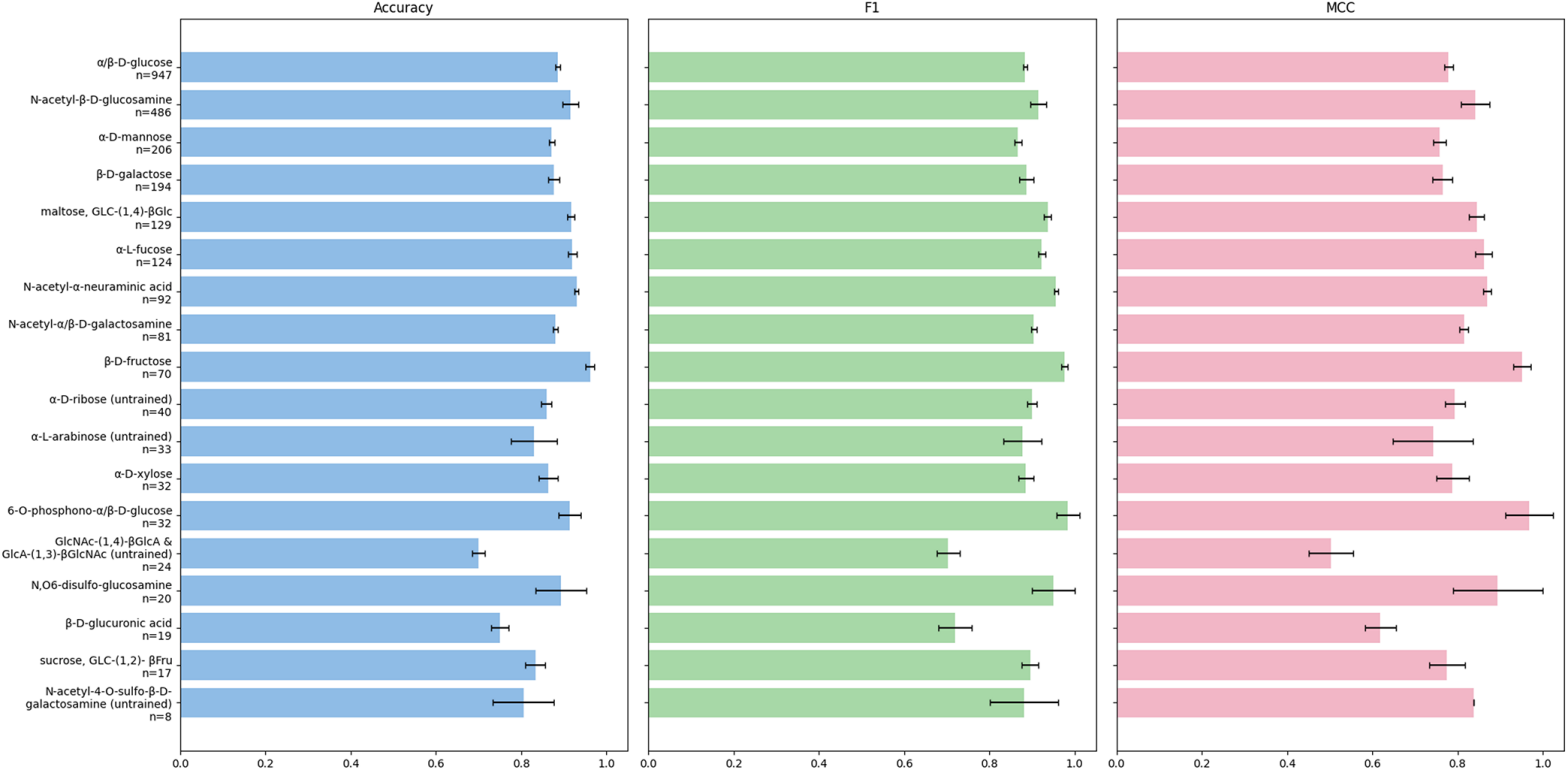
Accuracy, F1 score, and MCC of CLIMBS tested by different sugar–protein complexes. Fourteen types of sugar–protein complexes were used for training, and 4 types were not included in the training. For trained sugar types, the tested sample number is the size of the test set after splitting (1/5 of the whole data set). For untrained sugar types, the sample number is the size of the whole data set. Four models with different random seeds were used to assess performance variability.

The properties and usages of the CLIMBS model were further explored. The trained CLIMBS model can be retrained to include new sugar types while maintaining excellent performance when adding only tens of new samples to the training set. A robustness check (see Methods) revealed that both CH–π and polar interactions contribute to classification. A shortcut signal test (Methods) demonstrates that CLIMBS performance cannot be explained by simple graph-size statistics. Moreover, two case studies employing CLIMBS, focusing on carbohydrate–protein docking and carbohydrate binder design, were conducted to demonstrate the practical applicability of the model in carbohydrate–protein modeling.

### Performance Comparison with Other Methods

CLIMBS shows an outstanding performance in predicting sugar binding when compared to other scoring methods, according to the runtime and six model performance metrics (AUPRC, AUROC, accuracy, precision, specificity, and sensitivity) (Figure 3 and Supplementary Figure 3). A group of complexes in the test set was selected as the evaluation set for performance comparison (*db_eval*, see Supplementary Data 3j). We chose score functions from energy-based methods (Rosetta Energy Function REF,^12^ Autodock score function,^13^ HADDOCK score function^30^) and a confidence model from a machine learning approach (DIFFDOCK^18^).

**Figure 3:**
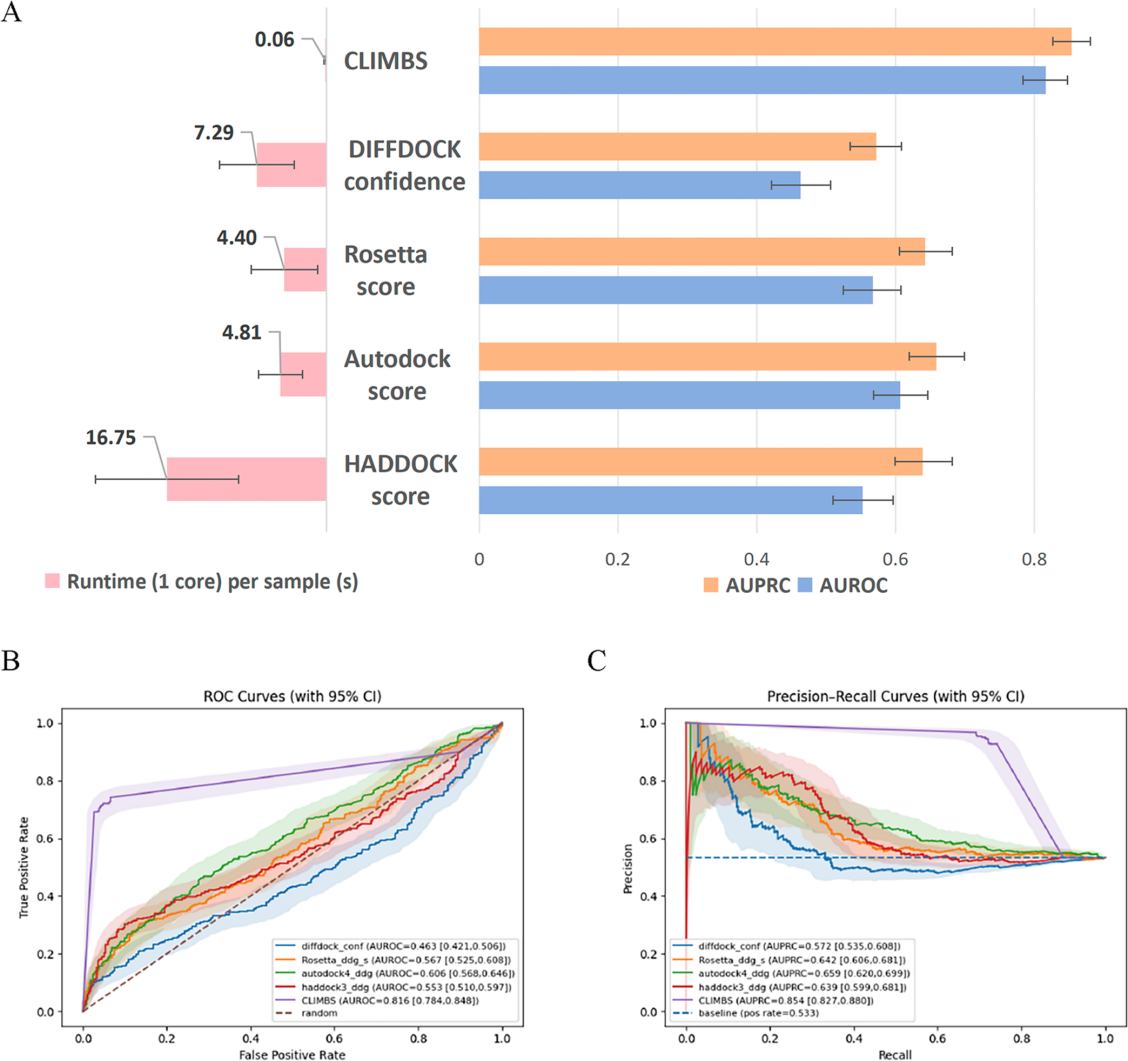
(A) Comparison between CLIMBS and other methods by different indicators: runtime per sample, AUPRC, and AUROC. (B) ROC curves and (C) PR curves of different methods with 95% confidence intervals. For the data set, see db_eval Supplementary Data 3j.

CLIMBS spends only 0.06s to predict one sample on one core. It is mainly because the classifier considers only the residues around the protein–sugar interface. CLIMBS has the best AUPRC of 0.816 and accuracy of 83.80%, which is more than 20% better than that of other methods compared. It also has a remarkable sensitivity of 74.02%, while other methods are under 40%. All five methods have higher specificity than sensitivity, indicating that the methods perform better on negative samples than on positive samples. CLIMBS has the smallest gap (19.02%) between specificity and sensitivity.

We then investigated the performance of the methods using the Wasserstein distance to quantify the difference between the distributions of positive and negative samples (Figure 4). CLIMBS’ predictions are separated, with a 0.670 Wasserstein distance. For the other four methods, the positive and negative distributions are overlapped in most cases, with 0.049 being the highest Wasserstein distance. Thus, CLIMBS can distinguish well between bound and nonbound samples within a diverse sugar binder data set.

**Figure 4:**
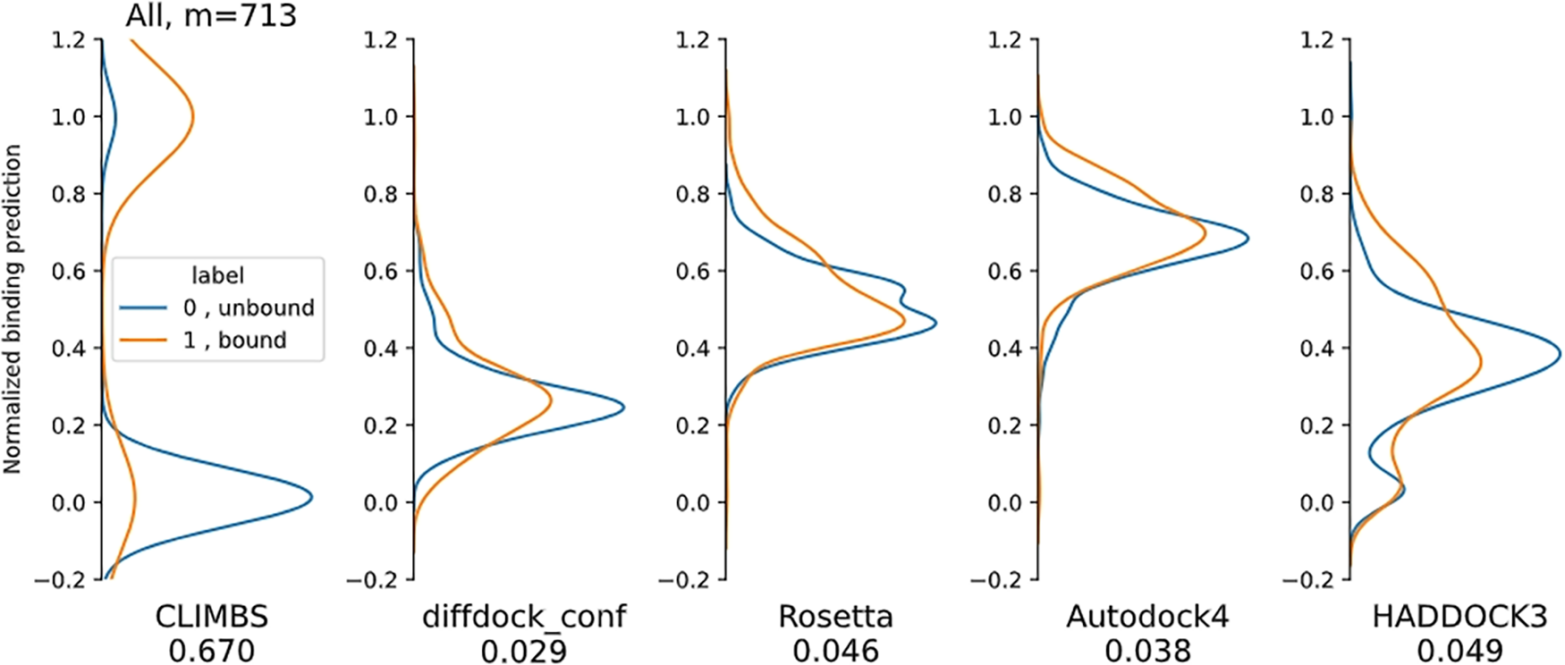
Comparison between CLIMBS and other methods by Wasserstein distance across multiple carbohydrates. Scores from each method were normalized between 0 and 1, corresponding to the lowest and highest scoring samples. Distributions of normalized scores are plotted as kernel density estimates. Positive samples are shown in orange and negative samples in blue. The number under each subplot is the Wasserstein distance between the normalized score distribution of positive/negative samples for each method. m: tested sample amount. For the data set, see db_eval Supplementary Data 3j.

### Sugar-Type Specificity

Due to the structural similarity of monosaccharides, the specificity of interacting sugar is an important performance indicator in modeling sugar–protein interactions. Therefore, we have evaluated CLIMBS sugar-type specificity on the carbohydrate-binding domain of *Marinomonas primoryensis* PA14 (MpPA14), which binds to a series of monosaccharides except N-acetyl-galactosamine (GalNAc). The apparent dissociation constants (K_d_app) of MpPA14-sugar complexes were measured by isothermal titration calorimetry (ITC) in previous work.^31^

Six solved complexes^31^ and one putative structure with GalNAc (generated by aligning and replacing GlcNAc with GalNAc in a solved MpPA14-GlcNAc complex) were selected as initial structures after Rosetta relaxation.^32^ Each initial structure underwent a Rosetta local docking protocol^33^ that generated 200 docked structures. CLIMBS was used to evaluate the binding of the initial and docked structures. Results in Table 1 show that the classifier, similarly to Rosetta Energy Function, can correctly identify that GalNAc is not binding while the other sugars are bound. However, for the best binding complex with fucose, the Rosetta Energy Function overestimates the binding free energy.

**Table 1:** Binding between MpPA14 and Different Sugars^a^

	experiment verification		crystallized structure		Rosetta local docking test	
sugar	Kdapp (mM)	ΔΔ*G*_298 K (kcal/mol)	CLIMBS	Rosetta ΔΔG (kcal/mol)	CLIMBS	Rosetta ΔΔG (kcal/mol)
l-fucose	0.65	–4.33	1	–7.08	0.90	–9.60 ± 0.76
N-acetyl-glucosamine	1.07	–4.04	1	–4.53	1.00	–3.34 ± 1.27
glucose	1.4	–3.88	1	–4.95	0.90	–3.07 ± 0.91
Mannose	1.7	–3.76	1	–3.75	1.00	–2.37 ± 0.77
l-arabinose	2.2	–3.61	1	–4.56	0.98	–0.03 ± 0.57
galactose	4.1	–3.24	1	–2.88	0.98	–1.89 ± 0.89
N-acetyl-galactosamine					0.30	4.81 ± 1.63

a Previous work^31^ measured the apparent dissociation constants (K_*d*_app) by isothermal titration calorimetry, and the binding free energy (ΔΔ*G*) was calculated based on K_*d*_app with a temperature of 298 K. Rosetta Energy Function and CLIMBS were used to estimate the binding of relaxed solved structures and the structure after running a Rosetta local docking. The local docking test values are the average and standard deviation of the results of 200 docking runs. For CLIMBS, the values represent the fraction of times that docking poses have been scored as positive. PDB ID of initial structure: 6X7J, 6X7Y, 6X8A, 6X7X, 6X8D, 6XAC.

To examine whether CLIMBS is capturing chemically meaningful carbohydrate features rather than memorizing generic pocket patterns, we performed a counterfactual analysis focusing on β-glucose complexes (Supplementary Data 11, Supplementary Figure 9). Perturbations included inversion or removal of individual hydroxyl groups (O1–O4), removal of the terminal O6 group, deletion of ring carbons, and complete removal of the sugar. CLIMBS predictions were highly sensitive to these chemically meaningful perturbations: removing the sugar converted all predictions to nonbinding, while removing ring carbons altered 21% of predictions, likely reflecting the loss of CH−π interactions. Orientation changes of hydroxyl groups generally produced stronger effects than their removal, suggesting that stereochemistry and polar interaction geometry are critical determinants of recognition. These results indicate that CLIMBS relies on stereochemical and functional-group features of carbohydrates rather than solely on protein pocket geometry, supporting its ability to distinguish closely related sugar types.

### Medium-Trained Model to Predict the Binding of Unseen Sugars

Several types of sugars have no solved structure in complex with binding protein, and CLIMBS is likely to encounter samples that include sugar types not used for training. To test the performance on unseen carbohydrates, a model (*db_p3*, see Supplementary Data 3d) trained on samples including various sugars except for GlcNAc-GlcA (NAG-BDP), arabinose (ARA), 5-O-phosphono-ribose (RP5), and 2-N-sulfo-6-O-sulfo-d-glucosamine (ASG) was used to test four unseen sugars with different training epochs (Figure 5). As the training epoch increases, the validation loss of trained sugar samples converges to 0.08 at 400 epochs. The loss of arabinose samples is comparable to that of trained samples, decreasing along with the training. However, the losses of the other untrained sugar samples stopped converging between 100 and 200 epochs. The loss of the untrained disaccharide GlcNAc-GlcA even begins increasing and reaches 0.29 at 450 epochs, while it is 0.13 at 275 epochs. The diverging losses of training and untrained sets indicate that overfitting of the model happens as the training epoch increases, indicating that a medium-trained model between 200 and 300 epochs is therefore an appropriate choice for predictions of untrained carbohydrates.

**Figure 5:**
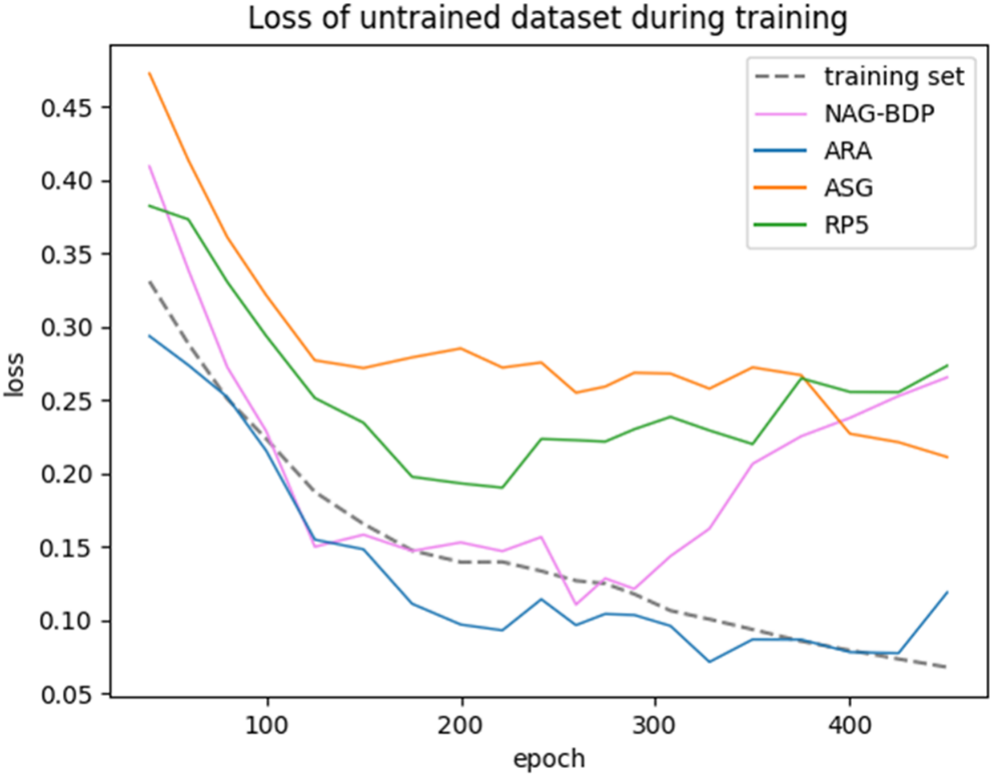
Loss curves of trained and untrained samples during CLIMBS training (see Supplementary Data 3d). The training set included 14 types of sugar binders (see Table 1). Four types of untrained samples are GlcNAc-GlcA (NAG-BDP), arabinose (ARA), 5-O-phosphono-ribose (RP5), and 2-N-sulfo-6-O-sulfo-d-glucosamine (ASG) binder.

### Retraining the Model for New Sugars

CLIMBS can be retrained for new sugar types with a limited number of samples to improve the performance on structures with novel sugars. Here, we assessed this with four retraining tests.

An initial database with samples containing proteins in complexes with highly represented carbohydrates in the PDB (Glc, GlcA, GlcNAc, galactose, fucose, mannose, and xylose in *db_r0*, see Supplementary Data 3e) was used to train a starting model. These seven sugars contain commonly shared fragments (e.g., central ring and hydroxyl groups). Our hypothesis was that due to the high structural similarity among monosaccharides, the accuracy for new carbohydrates could be rapidly increased with only a few samples introducing specific features. Pairs of additional carbohydrates that contain similar features and are absent from the initial data set were selected: one was added to the initial database for training and validation, and the second was used for monitoring accuracy improvement without training.

Two pyranoses, sialic acid and arabinose, were selected as first candidates with starting accuracy at 67.57% and 78.26%, respectively (Supplementary Figure 6A). The lower value for sialic acid might be due to a larger structural complexity (nine-carbon backbone) and the absence of an atom type (CH0 atom) in the database, while all atom types for arabinose are already present in the database. Sialic acid samples were progressively added to the training set, while arabinose was kept untrained (*db_r1*, see Supplementary Data 3f). For sialic acid, adding 12 samples is sufficient to reach an approximate 80% accuracy without decreasing the control accuracy (arabinose). The control accuracy dropped when more than 12 samples were added, indicating overfitting.

As the second case, we introduced fructose and ribose (Supplementary Figure 6B), which both contain a furanose ring not seen in the initial database (all trained carbohydrate types have a pyranose ring). The initial binding accuracy was high for ribose (88.24%) but not for fructose samples (56.52%). The lower value for fructose might be due to the absence of an atom type (CH0 atom) in the database. Then, fructose samples were added to the training set (*db_r2*, see Supplementary Data 3g). The accuracy of fructose samples increases significantly at 48 samples to 95.75%; however, it shows a small decrease in the ribose accuracy.

We also investigated how many samples were needed to retrain CLIMBS for new monosaccharides that include additional chemical modifications (Supplementary Figure 6C). Phosphorylation and sulfonation of carbohydrates are two frequently observed functional modifications that were not present in the initial database. N,O6-disulfo-glucosamine (SGN) and 6-O-phosphono-glucose (G6P) were selected for model training and testing, while N-acetyl-4-O-sulfo-galactosamine (ASG) and 5-O-phosphono-ribose (RP5) were selected only for testing (*db_r3*, see Supplementary Data 3h). Surprisingly, the initial model was not trained on phosphate, and sulfate has accuracies between 60% and 76% to predict the binding of modified sugars, likely driven by the rest of the carbohydrate structural features. Retraining with less than 40 samples with phosphorylated/sulfonated sugar improved binding accuracy to 76–85%, both for trained and untrained carbohydrates.

At last, we investigated retraining for disaccharides constructed by seen monosaccharides (Supplementary Figure 6D). Maltose (glucose-α-1,4-glucose) and sucrose (glucose-α-1,2-fructose) were selected as candidate disaccharides for training and testing (*db_r4*, see Supplementary Data 3i). Additionally, a heparan sulfate fragment, GlcNAc-GlcA (β-1,4 and β-1,3 linkage) disaccharide unit, was added for testing. The initial model, trained on only monosaccharide samples, has considerable performance on the disaccharide structures despite no glycosidic bond being included in the training set. The accuracy of sucrose and maltose increases from near 70% to 88.93% with 96 extra samples of sucrose and to 77.36% with 48 extra samples for maltose. The accuracy of GlcNAc-GlcA and repeat regions of unmodified heparan sulfate are hardly affected and fluctuate at a high value around 90%. It shows that the model trained by monosaccharides is able to assess disaccharides and even polysaccharides accurately. More generally, introducing 48 disaccharide samples into the training set can increase the accuracy before overfitting.

### Application to Protein Docking

CLIMBS can practically work as an additional evaluation method for carbohydrate–protein complex modeling problems. We applied it to a protein–carbohydrate global docking test to predict the correct placement of the ligand for the complexes. Structures in data set *db_dock* (Supplementary Data 3k) were used, including 197 samples with resolution lower than 2Å.

Docked conformations were generated with Chai-1.^34^ Only for 99 samples did the top-scoring model pass the structure-quality filters (see Supplementary Data 10), and these complexes were used for docking assessment.

We defined true positive docked structures as the poses with ligand RMSD < 2 Å after superposition of the protein model to the solved structure. CLIMBS, Rosetta score, and a CLIMBS + Rosetta combined metric were used to evaluate the binding state of the predicted structures. Models were considered positive (bound) for Rosetta score < −4.5 kcal/mol, capturing the majority of interactions observed in crystal structures for protein–carbohydrate complexes (see Supplementary Data 4). The CLIMBS + Rosetta metric scores a structure as bound when both CLIMBS and Rosetta score it as bound and evaluates it as nonbound when either CLIMBS or Rosetta scores it as nonbound. CLIMBS shows an accuracy of 58%, slightly higher than the Rosetta score (Figure 6). The CLIMBS + Rosetta combined metric performs similarly to CLIMBS alone, indicating that they discriminated complexes in a similar way. Within this test set, Rosetta’s sensitivity rises to 100% because no false negative samples are present with a ddG > −4.5 kcal/mol cut off, equivalent to *K*_d_ = 1 mM. Stricter energy cutoffs, such as −8 and −13 kcal/mol, improve accuracy for our test set (see Supplementary Data 10) but might be too strict for likely carbohydrate binders that operate in the low mM to high μM range.

**Figure 6:**
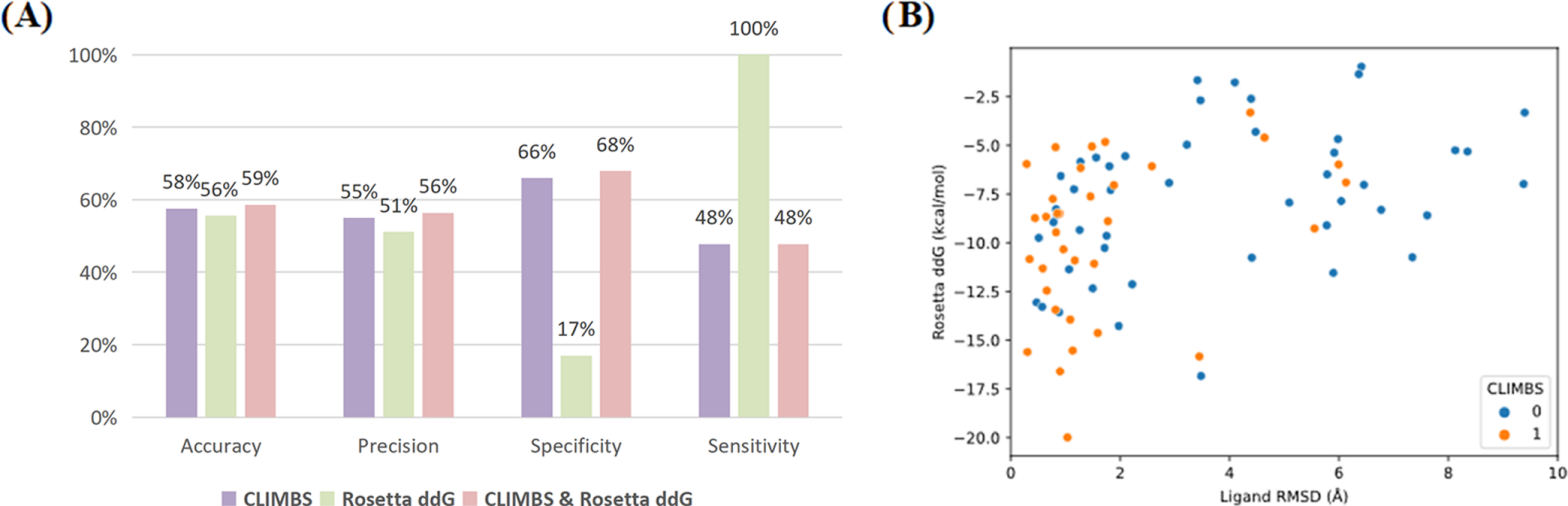
Scoring methods on carbohydrate–protein docking problem. Ninety-nine samples were included. Structures with ligand RMSD < 2 Å after superimposing the protein to the reference structure are regarded as bound. (A) Performance of each method. (B) Detail scatter plot for each carbohydrate–protein complex sample. Thresholds to predict as positive for each metric: CLIMBS = 1, Rosetta score function ddG < −4.5. For performance with different thresholds, Supplementary Data 10. The positive predictions of combined metrics are the AND subset of each positive prediction. accuracy: (TP + TN)/(TP + FP + TN + FN), precision: TP/(TP + FP), specificity: TN/(TN + FP), sensitivity: TP/(TP + FN). TP: True Positive, TN: True Negative, FP: False Positive, FN: False Negative.

### Application to Protein Design

CLIMBS can serve as a filter to identify promising binders during the carbohydrate-binding protein design. We assessed CLIMBS and Rosetta in evaluating a protein design task. A set of engineered carbohydrate-binding proteins developed through directed evolution from a nonbinding scaffold^35^ was used. Binding affinities and specificities have been experimentally determined between 4 protein variants and 13 carbohydrates, but no crystal structure is available. Bound protein–carbohydrate pairs were labeled as positives, while others were labeled as negatives (see Supplementary Data 10). All 52 complexes were predicted with Chai-1,^34^ and the top prediction was considered. The top prediction of only 37 generated structures passed the structure-quality filter (Supplementary Data 10) and were evaluated with CLIMBS, Rosetta, and the CLIMBS + Rosetta metric (Figure 7). CLIMBS shows 70% overall accuracy, while Rosetta reaches only 24%. CLIMBS + Rosetta shows the same accuracy as CLIMBS. Stricter cutoffs on the Rosetta score did improve the performance, resulting in 65% accuracy for the Rosetta score and 81% for the CLIMBS + Rosetta score (see Supplementary Figure 8). Overall, CLIMBS shows a reliable performance on the design problem, and the combination with Rosetta can improve the overall accuracy.

**Figure 7:**
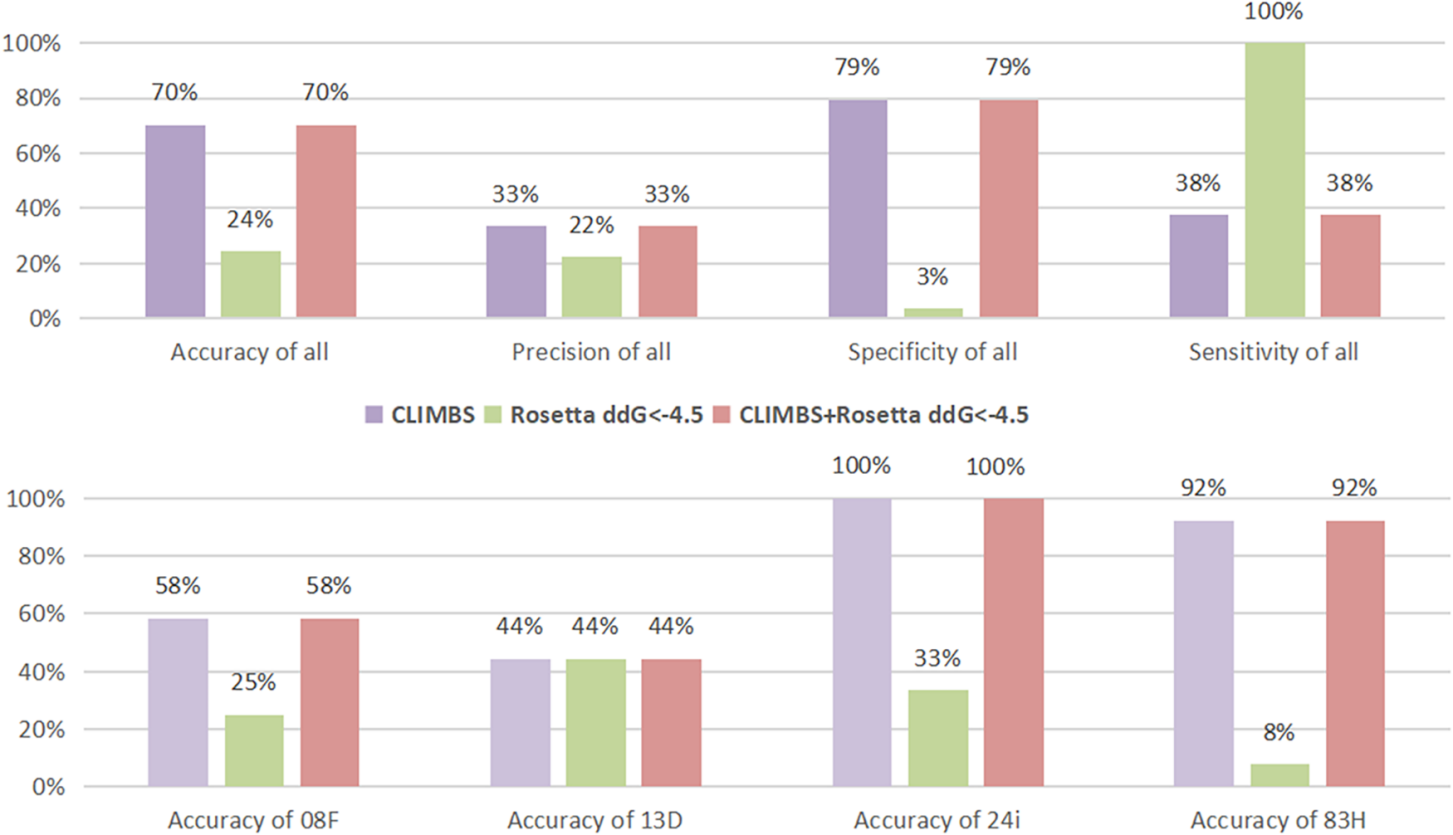
Performance of different scoring methods on the design carbohydrate-binding protein problem. In total, 37 samples were included: 12 samples of 08F, 9 samples of 13D, 3 samples of 24i, 13 samples of 83H. 08F, 13D, and 24i are binders with different specificities; 83H does not bind to any of the 13 targets analyzed. Four protein variants bind to different carbohydrates in 13 candidates. Thresholds to predict as positive for each metric: CLIMBS = 1, Rosetta score function ddG < −4.5. For performance with different thresholds, see Supplementary Data 10. The positive predictions of combined metrics are the AND subset of each positive prediction. accuracy: (TP + TN)/(TP + FP + TN + FN), precision: TP/(TP + FP), specificity: TN/(TN + FP), sensitivity: TP/(TP + FN). TP: True Positive, TN: True Negative, FP: False Positive, FN: False Negative.

## Discussion

In this study, we have developed CLIMBS, a machine learning classifier that assesses the binding state of carbohydrate–protein complex structures.

A sugar–protein complex library was built to include 19 different carbohydrates for model training and testing. Experimentally solved sugar–protein complexes and failed carbohydrate binder designs were collected and labeled as positive and negative samples, respectively, while other methods^20,21^ tend to use solved binder structures with scalar labels such as experimental binding affinity. Binary labels were chosen because there is no scalar experimental result for nonbinding and, within a protein design pipeline framework, a primary focus would be on identifying nonbinding complexes that should not be taken into the next steps.

Using binary labels allows CLIMBS, on one hand, to effectively discriminate between good-binding and poor or nonbinding complexes. This makes it a method independent of and unrelated to conventional score functions and confidence metrics. On the other hand, the binary setup also means that CLIMBS cannot directly describe the strength or extent of sugar–protein interactions. Nevertheless, in cases such as validation of multiple local docking poses, a crude but effective scalar value can be obtained by averaging the scores across the entire population.

The use of negative data originating from a computational protein design pipeline is a distinctive feature of our approach to classifier training. Selecting suboptimal carbohydrate binder designs as negative data was a key element in constructing the training data set and contributed significantly to the performance of CLIMBS. This strategy enabled us to build a balanced data set and to focus training on atomic details specific to carbohydrate–protein interactions. It also highlights the power of synthetic data augmentation to overcome the limitations of current scoring functions, which were originally used to design these structures.

The generalizable architecture of CLIMBS for protein–carbohydrate interactions enables it to address sugars not included in the training set. Its fragment-level representation preserves essential information among atomic functional groups, facilitating the learning of the features that are present in multiple molecules (e.g., the carboxyl group in aspartic acid, glutamic acid, and glucuronic acid). Potential overfitting can be mitigated by using lower-epoch models, and the novel sample performance can be improved by retraining with a small number of additional samples. These characteristics make the CLIMBS framework particularly well-suited for carbohydrate–protein studies, where available data sets are relatively small and different carbohydrates share similar structural motifs, thus enhancing the recognition of common features.

CLIMBS comprehensively considers interactions between the carbohydrate and protein. CH-π interactions, which are often fundamental to sugar binding but are neglected by most energy-based methods, are included as a key type of interaction in this model. Metal ions are considered implicitly in CLIMBS. There are 141 carbohydrate–protein complexes in the library containing at least one metal atom in the binding site. Although the metal ions were removed during the data preprocessing, the residues interacting with the metal ions were kept and provided with this information during training. Similarly, apolar interactions disfavored by sugars and steric clashes are implicitly addressed during relaxation in the structure preprocessing step.

Compared to other score functions and machine learning methods, CLIMBS has shown outstanding performance in the recognition of bound samples. As an entirely geometry-based method, it can distinguish different sugars with highly similar structures that energy-based methods cannot. The subsecond runtime makes it feasible to combine with current energy-based methods or machine learning methods, filtering false positive/negative results, and increasing the overall pipeline accuracy. It is therefore a new scoring tool, orthogonal to other energy and machine learning-based methods, that, when combined with docking and design protocols and pipelines, allows for improving the selection of protein–carbohydrate models that retain native-like features.

On practical applications such as scoring of docking and design, the accuracy of CLIMBS decreases, as expected, since the tasks differ slightly from the training sets and are more challenging. CLIMBS was trained to distinguish native bound structures and artificially generated nonbound structures, although those are often energetically similar. However, in realistic docking and design cases, positive and negative samples are usually not known and are produced using the same method (generative model followed by relaxation in our case). The quality of samples passed to CLIMBS is also critical: only about half of the generated models were acceptable in terms of quality metrics (protein and ligand pLDDT), and this can bias the range of complexes analyzed, with a chance of potentially losing weaker binders.

Training CLIMBS on a restricted data set containing only high-resolution structures (<2 Å) (data set *db_w2*, Supplementary Data 3l) resulted in a model able to provide higher discrimination (64% accuracy up from 58%) in docking assays where the test set was also composed only of high-resolution structures of complexes, likely to have high affinity. However, in practical cases where binding affinity is likely in the millimolar or high micromolar range, such as in the design cases, the general model still performs best, and the performance can be further boosted by combining it with Rosetta scoring.

CLIMBS is characterized by architectures and embeddings that leverage shared structural features of carbohydrates and by training with negative synthetic data that have driven its discrimination power. This approach can be potentially generalized to other supervised learning methods, making use of this large untapped resource of data.

## Methods

### Model Architecture

CLIMBS takes PDB structure files containing a carbohydrate chain and one or more protein chains as input and outputs values of 1 (binding) or 0 (not binding). Protein–sugar interacting graphs are generated from data preprocessing. Then, the interacting graphs are passed to the embedding module, including spherical message passing layers,^36^ principal neighborhood aggregation layers,^37^ and a supernode-based hierarchical graph pooling layer.^38^ Followed by fully connected layers and a sigmoid function, binary results are returned (Supplementary Data 5).

Data preprocessing elicits the essential details of protein–sugar interactions in the PDB structure file. First, it selects the amino acid and carbohydrate residues on the interface. Only the amino acids/saccharides that have polar or CH-π interaction with the saccharide/amino acids are kept (see Supplementary Data 1). Hydrogen atoms are discarded. Second, it elicits the minimal binding site from the interface residues: for amino acids, either the terminal functional groups of side chains or the mainchain backbones are kept; for saccharides, all heavy atoms are kept. Finally, the information on the minimal binding site is generated, including atom coordinates, an atom-level interacting graph *G = {V,E}*, and an assignment matrix *S*. Node matrix *V* includes the one-hot-encoded atom types. Edge matrix *E* describes three types of interactions between nodes: chemical bond, polar interaction, and CH-π interaction. Assignment matrix *S* assigns atoms into the corresponding fragments. Fragments are groups of atoms that commonly exist in different molecules. For example, glucose is constructed by a six-member pyranose ring fragment, 4 oxygen fragments, and a carbon–oxygen (single bond) fragment. The fragment-level pooling layer of CLIMBS can capture the features of fragments shared among different types of sugars.

Given the preprocessed data, the classifier model returns binary values indicating bound or unbound. In the beginning, the interaction graph *G = {V,E}* as well as the atom coordinates are passed to spherical message passing (SMP) layers^36^ to generate an updated graph *G′* = *{h,E*′*}.* The first SMP layer converts the atom coordinates into three geometrical representations (distances, angles, and torsions) and incorporates them into the interaction graph *G*. Then, the rest of the SMP layers update edges and aggregate them into the node feature matrix *h*. Next, principal neighborhood aggregation (PNA)^37^ layers update *G*′ *= {h,E*′*}* into *G*″ *= {h*′*,E*′*}* by a multiple aggregation function and degree-scalers. After that, a pooling layer coarsens the atom-level graph *G*″ *= {h*′*,E*′*}* into a fragment-level graph G* = {h*,E*} with the assignment matrix *S*. Then, fragment-level node feature h* is passed to a sum aggregator and an activation function to output global features *u*. The global features are then passed to fully connected layers and a sigmoid function to output normalized predictions *p* ∈ [0,1]. Finally, 0 or 1 is outputted by comparing the *p* to a threshold value (default 0.5, for details see Supplementary Data 5) to indicate whether it is unbound or bound.

### Data Sets

Positive samples were derived from the Protein Data Bank.^22^ Structures were validated (Supplementary Data 4) to ensure that high-quality structures are used. To increase reliability and accuracy, redundant cleaning was done for the positive database according to the protein–sugar complex RMSD and the interface RMSD. All positive samples were relaxed by Rosetta^39^ to alleviate potential clashes.

Negative samples were obtained from a computational design. Experimental success rate of designed binders for small molecules is around 0.67% according to the latest RFAA design method,^24^ though with high scoring for energy- and structure-based metrics, e.g., AF2 pLDDT >80, designed RMSD < 2 Å, low Rosetta ddG (<−30 in the case of digoxigenin binder), and at least one hydrogen bond to ligand. Therefore, poorly scoring designs can be reasonably assumed as failures. Here, a Rosetta design protocol (see Supplementary Data 4) was used to generate carbohydrate–protein complexes. The top 10% worst (ranked by binding free energy of Rosetta Energy Function) complexes were selected as negative samples. In general, those sugar binders underperform because of either a lack of interactions (e.g., cavities, shallow clefts) or inaccurate interactions (e.g., clashes and distorted hydrogen bonds).

### Model Optimization

The training of CLIMBS was supervised by the binary 0/1 label of the negative and positive samples. Mean absolute errors (MAE, also known as L1 loss) between labels and predictions *p* were used for parameter optimization. The sugar–protein complex structure library comprises a ratio of positive and negative samples which is approximately 1:1. Fifteen types of monosaccharides, 3 types of disaccharides, and a linear oligosaccharide with the corresponding binders, in total 13,507 samples are in the library (see Supplementary Data 3a). The library was split into training, validation, and test sets with the ratio of 2:2:1, while maintaining the 1:1 positive and negative ratio (for training detail, see Supplementary Data 2).

### Pooling Layer Selection

We have explored three representations of minimal binding sites as pooling layers according to the scale of molecular structure detail: atom-, fragment-, and molecule-level representations. Classifier models with those three different pooling layers before the sum aggregator were trained and tested (Results in Supplementary Data 6), with the fragment-level pooling layer performing best for both monosaccharide prediction and disaccharide prediction.

### Robustness Check

A robustness check was performed to unravel the factors contributing to CLIMBS’ performance. Model robustness is the ability of the model to maintain performance when it faces adversarial data compared with the training data. Adversarial samples were generated by slightly modifying the original samples, such as with masked edges and extra nodes or edges in the interacting map G. Results in Supplementary Data 7 reveal that CH-π interacting residues increase the probability of prediction as positive (bound), but are not the only factor, consistent with experimental results from Hudson.^40^

### Shortcut Signal Test

To assess whether preprocessing introduces shortcut signals, we evaluated simple baseline models using graph-related feature counts (Results in Supplementary Data 12). Baselines based on simple graphic features counts (e.g., atoms or polar interactions) showed limited predictive power and performed substantially worse than CLIMBS. This result indicates that the model’s performance cannot be explained by simple graph-size statistics or other coarse structural counts, suggesting that CLIMBS instead learns more informative structural patterns underlying carbohydrate–protein interactions.

## Supplementary Material



## Data Availability

The code and CLIMBS model are available as part of the Rosetta ML package (https://github.com/Parmeggiani-Lab/sugar_binding_predictor2). The data set of positive and negative samples is available in Zenodo (https://zenodo.org/records/15169999). The protocol used to design negative sample is documented in https://github.com/Jacky233emm/new_residue_design.

## Notes

Additional model details, analysis, data and methods, including Supplementary Figures 1–11 and Supplementary Tables 1–5 (PDF)
