# Supplementary material for: CLIMBS: Assessing Carbohydrate–Protein Interactions through a Graph Neural Network Classifier Using Synthetic Negative Data
Source: J Chem Inf Model. 2026 Apr 3;66(11):6271–80. doi: 10.1021/acs.jcim.6c00126 (PMC13250977; doi:10.1021/acs.jcim.6c00126)
Supplement: Supplementary file 1 [file ci6c00126_si_001.pdf]

Supporting Information for

# CLIMBS: assessing Carbohydrate-Protein interactions through a graph neural network classifier using synthetic negative data

**Authors:** Yijie Luo[1*], Fabio Parmeggiani,[1,2,3*]

**Affiliations:**

[1]School of Biochemistry, University of Bristol; University Walk, Bristol BS8 1TD, UK.

[2]School of Chemistry, University of Bristol; Cantock's Close, Bristol BS8 1TS, UK.

[3]School of Pharmacy and Pharmaceutical Sciences, Cardiff University, Redwood Building, King Edward VII Ave, Cardiff, CF10 3NB, UK

*Corresponding authors. Email: yijie.luo@bristol.ac.uk , parmeggianif@cardiff.ac.uk

Supporting Information for ........ 1

Supplementary Data 1. Definition of polar interaction and CH-π interaction ........ 2

Supplementary Data 2. Model optimization ........ 2

Supplementary Data 3. Dataset detail for the used models ........ 3

Supplementary Data 4. Processing positive samples and generating negative samples 7

Supplementary Data 5. Model architecture ........ 9

Supplementary Data 6. Pooling layer selection ........ 9

Supplementary Data 7. Robustness check ........ 10

Supplementary Data 8. Calibrating other methods ........11

Supplementary Data 9. Details of new sugar retraining ........ 14

Supplementary Data 10. CLIMBS on docking and design problems ........................... 15
Supplementary Data 11. Counterfactual test ................................................................. 18
Supplementary Data 12. Graph statistics and shortcut signal tests ................................ 20
Supplementary method 1. Rosetta relaxation and docking(14) ..................................... 22
References ........................................................................................................................ 27

*Supplementary Data 1. Definition of polar interaction and CH-π interaction*

Polar interactions were defined as contacts between N or O atoms from two different molecules within 3.5 Å. CH-π interactions were defined according to previous research (*1*). In short, a CH-π interaction exists between the CH group of the sugar ring and aromatic amino acids, limited by the distance (<4.5 Å) of C to the center of the aromatic ring, the angle between the CH vector and the aromatic ring normal (<40°), and the distance between the C projection on the aromatic ring and center of the aromatic ring (<2 Å).

*Supplementary Data 2. Model optimization*

Datasets for model optimization were divided into training, validation, and test in a 2:2:1 ratio. Models whose validation loss remained lower than the training loss for more than ten consecutive epochs were considered to have completed training. The maximum training epoch was 500. The performance (e.g., accuracy and specificity) of trained models was tested on the test set.

Each negative carbohydrate-protein complex sample generated by Rosetta(*2*) includes a CH-π interacting residue to the binding sugar. However, only around 30% of the native binders from RCSB(*3*) (positive sample) had CH-π interaction with sugars. Pre-testing had shown that using original negative samples for training would lead to severe bias in the CH-π interaction. Thus, only 30% of the negative samples would keep the CH-π interacting residue during data pre-processing, while in the remaining 70%, the CH-π-interacting residue was mutated to alanine.

During model training, merged samples were used to reduce the bias between complexes, including monosaccharide and more than one carbohydrate unit. Merged samples were generated by combining two different structure files with monosaccharides into one structure file. Only all-positive or all-negative merged samples were used.

*Supplementary Data 3. Dataset detail for the used models*

This section states the components of the datasets for the models used. Complexes with 23 different ligands were collected in the library(*4*) (supplementary table 1).

*Supplementary Table 1 Ligand type involved in the library of carbohydrate-protein complexes.*

| Carbohydrate type in complex | Ligand ID |
|---|---|
| N-acetyl-α-D-galactosamine | A2G |
| α-L-arabinose | ARA |
| N-acetyl-4-O-sulfo-β-D-galactosamine | ASG |
| β-D-glucuronic acid | BDP |
| 6-O-phosphono-β-D-glucose | BG6 |
| β-D-glucose | BGC |
| GlcNAc-(1,4)-βGlcA & βGlcA-(1,3)-GlcNAc | di12 |
| maltose | di3 |
| sucrose | di4 |
| β-D-fructose | FRU |

| | |
|---|---|
| α-L-fucose | FUC |
| 6-O-phosphono-α-D-glucose | G6P |
| β-D-galactose | GAL |
| α-D-glucose | GLC |
| α-D-mannose | MAN |
| N-acetyl-β-D-glucosamine | NAG |
| N-acetyl-β-D-galactosamine | NGA |
| Repeating region of heparan sulphate (constructed by 3 GlcA and 3 GlcNAc) | poly1 |
| α-D-ribose | RIB |
| 5-O-phosphono-β-D-ribose | RP5 |
| N,O6-disulfo-glucosamine | SGN |
| N-acetyl-α-neuraminic acid | SIA |
| α-D-xylose | XYS |

As mentioned in Supple, there are training, validation, and test sets in the model optimization dataset. Some models used extra datasets to evaluate the properties of the model. Samples in one type of ligand were used for training/validation/test sets with a ratio of 2:2:1 in model optimization, for validation/test set, a ratio of 2:1 in extra evaluation, or for a 100% test set in testing only.

a. Dataset for final model (*db_w1*):

Complexes with ligand A2G, BDP, BG6, BGC, di3, di4, FRU, FUC, G6P, GAL, GLC, MAN, NAG, NGA, SGN, SIA, and XYS were used for model optimization. Complexes with ligand RIB, ARA, ASG, di12, and poly1 were for testing only.

b. Dataset for pooling layer optimization (*db_p1*):

Complexes with ligand NAG, BDP, and di12 were used for model optimization.

c. Dataset for robustness check (*db_p2*):

Complexes with ligand NAG and BDP were used for model optimization.

Complexes with ligand GLC were for testing only.

d. Dataset for predicting unseen carbohydrates (*db_p3*):

Complexes with ligand GLC, NAG, BDP, BGC, GAL, FUC, MAN, SIA, XYS, and FRU were used for model optimization. Complexes with ligand di12, ARA, ASG, and RP5 were for testing only.

e. Dataset for new sugars retraining (initial, *db_r0*):

Complexes with ligand GLC, NAG, BDP, BGC, GAL, FUC, MAN, and XYS were used for model optimization.

f. Dataset for new sugars retraining (unseen monosaccharide, *db_r1*):

Complexes with ligand GLC, NAG, BDP, BGC, GAL, FUC, MAN, and XYS were used for model optimization. Complexes with ligand ARA, SIA were used for extra evaluation. Different numbers of SIA (0,12,24,48 samples) are added to the training set.

g. Dataset for new sugars retraining (unseen furanose ring, *db_r2*):

Complexes with ligand GLC, NAG, BDP, BGC, GAL, FUC, MAN, and XYS were used for model optimization. Complexes with ligand RIB, FRU were used for extra evaluation. Different numbers of FRU (0,12,24,48 samples) are added to the training set.

h. Dataset for new sugars retraining (unseen fragment, *db_r3*):

Complexes with ligand GLC, NAG, BDP, BGC, GAL, FUC, MAN, and XYS were used for model optimization. Complexes with ligand RP5, G6P, ASG, and SGN were used for extra evaluation. Different numbers of G6P (0,32 samples) as

well as SGN (0,37 samples) samples are added to the training set.

i. Dataset for new sugars retraining (unseen disaccharide, *db_r4*):

Complexes with ligand GLC, NAG, BDP, BGC, GAL, FUC, MAN, and XYS were used for model optimization. Complexes with ligand di12, di3, and di4 were used for extra evaluation. Different numbers of di3 (0,6,12,24,48,53 samples) as well as di4 (0,6,12,24,48,60 samples) samples are added to the training set.

j. Dataset for methods' comparison (*db_eval*):

Here is the final model (Supplementary Data 3a). Dataset for model performance evaluating samples in the testing set of NAG, GLC, BGC, GAL, FRU, di3, and di4 after splitting.

k. Dataset for docking application evaluation (*db_dock*):

It includes 197 samples in the testing set with PDB structure resolution<2 Å, containing ligand XYS, SIA, NGA, NAG, MAN, GLC, GAL, G6P, FUC, FRU, BGC, BG6, BDP, ASG, ARA, A2G.

l. Dataset for re-training model to improve performance on docking application (*db_w2*):

High PDB structure resolution (<2 Å) complexes with ligand A2G, BDP, BG6, BGC, di3, di4, FRU, FUC, G6P, GAL, GLC, MAN, NAG, NGA, SGN, SIA, and XYS were used for model optimization. Complexes with ligand RIB, ARA, ASG, di12, and poly1 were for testing only.

*Supplementary Data 4. Processing positive samples and generating negative samples*

Positive samples were experimentally solved structures of native sugar-binding proteins from the Protein Data Bank. Only high-quality structures were retained. Structure quality was assessed by PDB resolution (<3Å), atom occupancy (>0.9), and B-factor (<80) (*5*). Ligand structure quality was further assessed by RSCC (>0.8), RSR (<0.3), bond RMSZ (<2), and angle RMSZ (<2) (*6*). Each target sugar and nearby (within 4.5Å for aromatic residue and 3.5 Å for other) protein chain(s) were saved as a sugar-protein complex. Complexes with the same ligand, sequence identity >25% and binding site (sugar around 4.5Å) RMSD <1Å were regarded as redundant; only one of them would be kept. Then, the complexes were relaxed with coordination-constrained by Rosetta. To strip out the samples with low binding affinity, filters including interface residue number >4 and Rosetta ddG <-4.5 kcal/mol (approximately dissociation constant 0.5 mM). The ΔΔG cutoff is close to the binding free energy value in native carbohydrate binding protein (*7*), and also near the peak of Rosetta ddG distribution of positive samples before filtering.

*Supplementary Figure 1 Rosetta ddG distribution of positive samples after Rosetta relaxation and before filtering. 90% of the sample has ddG from -0.69 to -19.69 (allowance range).*

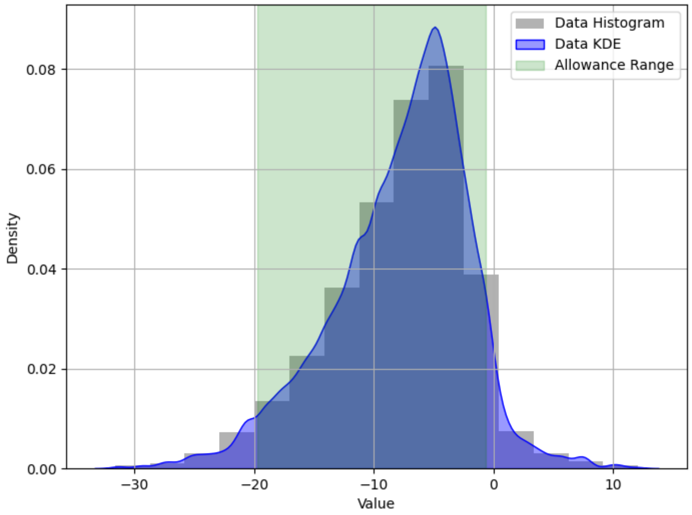

Negative samples were generated by computational design. A Rosetta design protocol named *new residue design* is used to generate sugar-binding proteins that include CH-π interactions. In stage 1, it finds a suitable pocket for a ligand in a given protein scaffold and alters the interacting residue to alanine. Then, it designs a protein sequence to have proper polar and CH-π interactions to target sugar in stage 2. Selecting the top 10% of the worst-designed proteins in both stage 1 and stage 2 according to Rosetta binding free energy. Those would be the negative binder database, with either a lack of interaction or inappropriate interaction with sugar. Comparison between positive and negative samples is shown in Supplementary Figure 2.

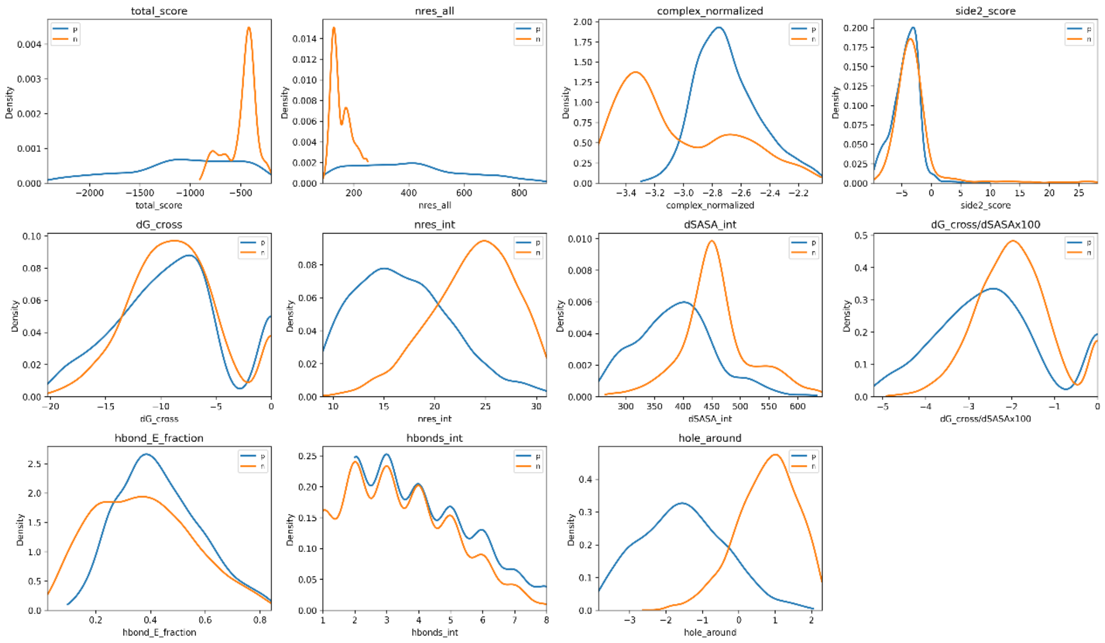


*Supplementary Figure 2: Structural analysis between positive and negative structures for CLIMBS with 11 Rosetta metrics. 3920 samples were included. total_score: Rosetta score of the complex; nres_all: residue count of the complex; complex_normalized: Average energy of a residue in the entire complex; dG_cross: binding free energy (ΔΔG) between protein and sugar in the complex; nres_int: interface residue count; dSASA_int: interface SASA; dG_cross/dSASAx100: binding free energy per unit interface area * 100; hbond_E_fraction: Amount of binding free energy accounted for by cross interface hydrogen bonds; hbonds_int: cross interface hydrogen bond count; hole_around: degree of voids around the sugar in the complex. A value of 0.0 means on par with native structures observed in the PDB; positive is worse (more voids), negative is better (less voids).*

## *Supplementary Data 5. Model architecture*

*Supplementary Figure 3: The architecture of the CLIMBS classifier model*

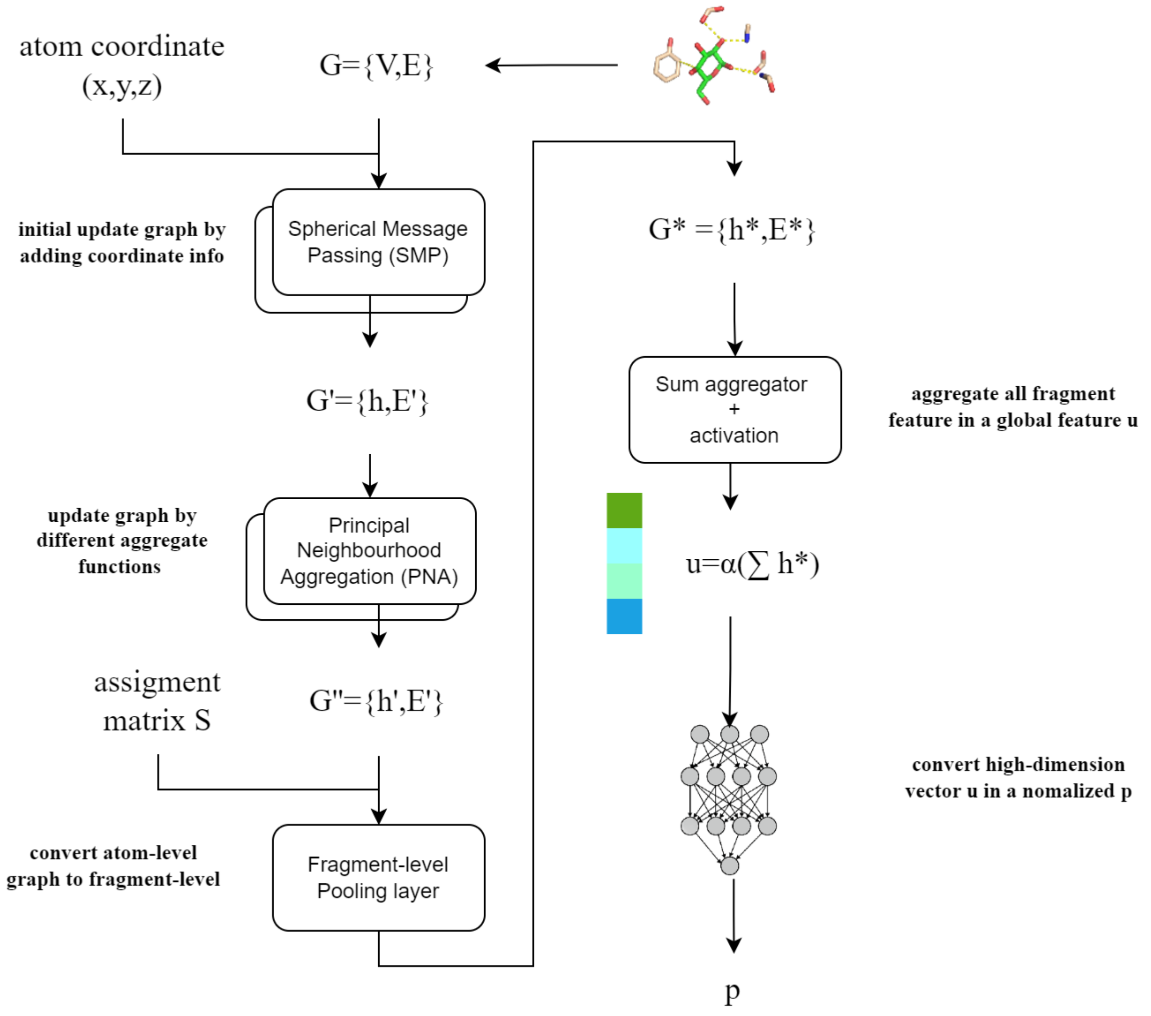


*The minimum binding site information is input to the model, including atom coordinates of the structure, an interaction graph G, and an atom-to-fragment assignment matrix S. After several blocks (SMP, PNA, pooling layers, and sum aggregator), the classifier model outputs a normalized p. The activation function (α) is a rectified linear unit (ReLU) in the model.*

## *Supplementary Data 6. Pooling layer selection*

As a result, in Supplementary Table 2, all three models have similar performance for monosaccharide samples prediction, with the highest accuracy of 99.52% for the fragment-level model and the lowest accuracy of 96.38% for the molecule-level model.

For disaccharide samples prediction, the fragment-level model is apparently better than the other, with an accuracy of 81.25% that 6.25% and 12.50% higher than molecule-level and atom-level, respectively.

*Supplementary Table 2: Accuracy and training loss of CLIMBS with different pooling layers. Classifiers here were trained on GlcNAc, GlcA, and GlcNAc-GlcA (db_p2, Supplementary Data 3b). The 2$^{nd}$ and 3$^{rd}$ columns are the testing results on GlcNAc and GlcA, the 4$^{th}$ and 5th columns are on GlcNAc-GlcA disaccharides.*

| Pooling layer | monosac loss | monosac accuracy | disac loss | disac accuracy |
|---|---|---|---|---|
| atom-level | 0.022 | 98.07% | 0.277 | 68.75% |
| fragment-level | 0.019 | 99.52% | 0.19 | 81.25% |
| molecule-level | 0.056 | 96.38% | 0.245 | 75.00% |

*Supplementary Data 7. Robustness check*

From the pooling layer selection assay, we knew CLIMBS performed quite well on trained sugar type samples. To make the model more fragile and sensitive during evaluation, a model that was trained on GlcNAc and GlcA was used to evaluate glucose (Glc) (for details see Supplementary Data 3c). The model had an initial accuracy of 76.36% (Supplementary Table 4), but different disturbances were introduced to the test set. Missing the CH-π interacting information has almost no effect, but missing all interaction information or removing the CH-π interacting residue decreases 4% of the accuracy, with a drop in true positive predictions. Moreover, adding an additional CH-π interacting residue significantly decreases the amount of true negatives with an accuracy drop of -19.17%. Although the disturbances of the CH-π residue harm the CLIMBS performance, the accuracies of the attacked samples (72.00% and 57.19%) are still higher than 50%.

*Supplementary Table 3: Accuracy and training loss of CLIMBS facing adversarial attacks. The model here was trained on GlcNAc and GlcA, and tested by Glc samples with missing or additional information (db_p2, see Supplementary Data 3c). TP: true positive, FN: false negative, TN: true negative, FP: false positive.*

| Disturbance | Loss | Accuracy | TP | FN | TN | FP |
|---|---|---|---|---|---|---|
| -- | 0.297 | 76.36% | 403 | 57 | 298 | 162 |
| remove CH-π interactions | 0.299 | 76.47% | 404 | 56 | 298 | 162 |
| remove all interactions | 0.319 | 72.22% | 368 | 92 | 295 | 165 |
| remove CH-π residues in the positive sample | 0.315 | 72.00% | 363 | 97 | 298 | 162 |
| Add CH-π residues in the negative sample | 0.429 | 57.19% | 403 | 57 | 122 | 338 |
| | | | 460 | | 460 | |

## *Supplementary Data 8. Calibrating other methods*

Every method needs to set a threshold value to separate predicted binding and unbound, since Rosetta Energy Function(*8*), Autodock4 score function(*9*), HADDOCK score function(*10*), and DIFFDOCK(*11*) confidence model return a scalar value rather than a binary value.

We investigated the effect of threshold value on model accuracy, sensitivity, specificity, precision, and F1 score of all methods. To get the best performance of each model, the threshold value that tuned on validation test and has the maximum accuracy was chosen (supplementary table 5 and supplementary figure 4).

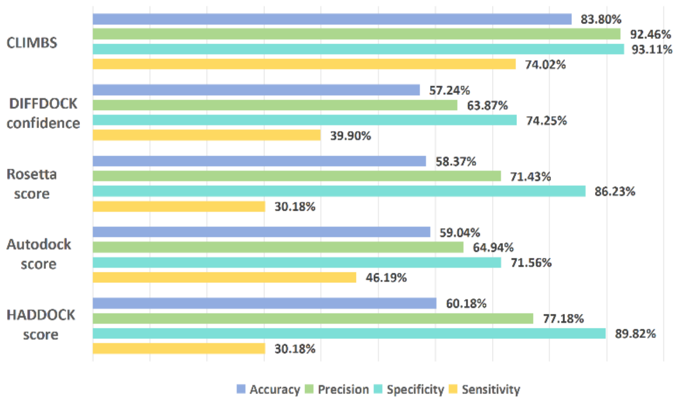


*Supplementary Figure 4 Comparison between CLIMBS and other methods by different indicators: runtime per sample, accuracy: (TP+TN)/(TP+FP+TN+FN), precision: TP/(TP+FP), specificity: TN/(TN+FP), and sensitivity: TP/(TP+FN). TP True Positive, TN True Negative, FP False Positive, FN False Negative. Thresholds of predicting as bound for each metric are in Supplementary Table 5 and Supplementary Figure 4*

*Supplementary Table 4 Threshold and normalised threshold value of each method to predict as bound.*

| | Threshold | Normalised threshold |
|---|---|---|
| CLIMBS | >0. 0159 | >0. 0159 |
| Rosetta binding free energy | <-12. 9889 kcal/mol | <0. 597 |
| Autodock4 binding free energy | <-3. 5982 kcal/mol | <0. 7188 |
| HADDOCK3 binding free energy | <-26. 2725 kcal/mol | <0. 4908 |
| DIFFDOCK confidence | <-3. 3255 | <0. 3008 |

*Supplementary figure 5 Normalised score calibration for positive sample and negative sample for CLIMBS and other methods.*

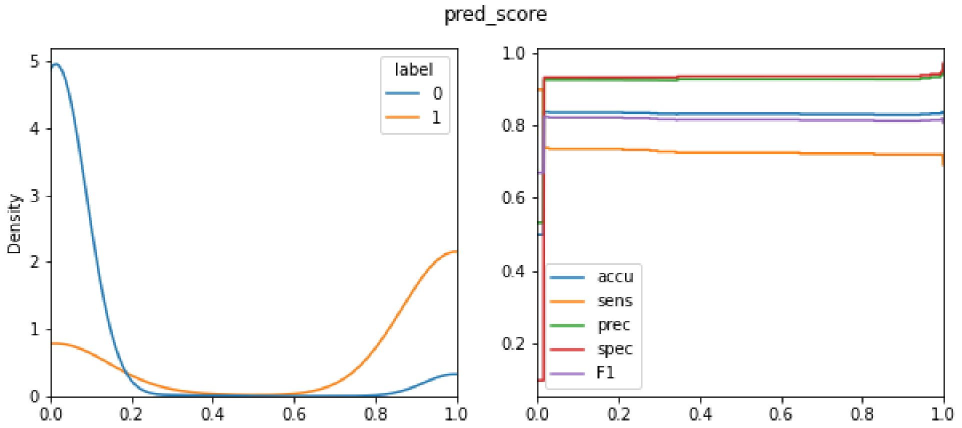

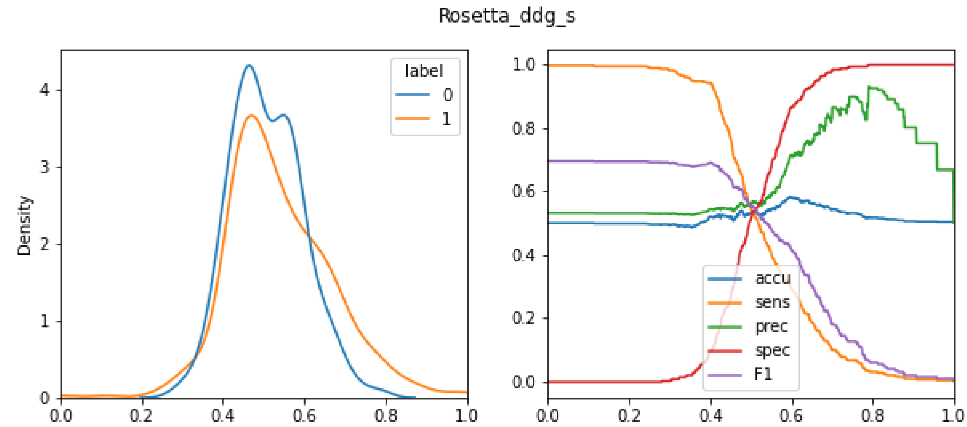
Rosetta_ddg_s
Density
label
0
1
accu
sens
prec
spec
F1

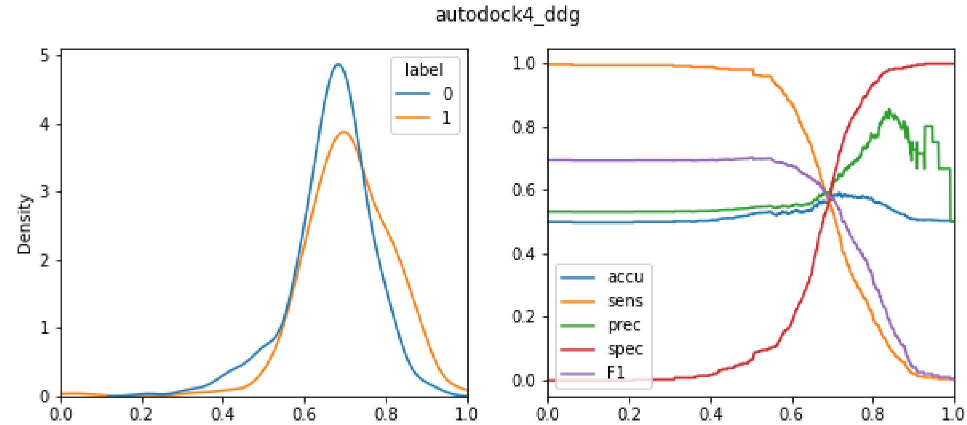
autodock4_ddg
Density
label
0
1
accu
sens
prec
spec
F1

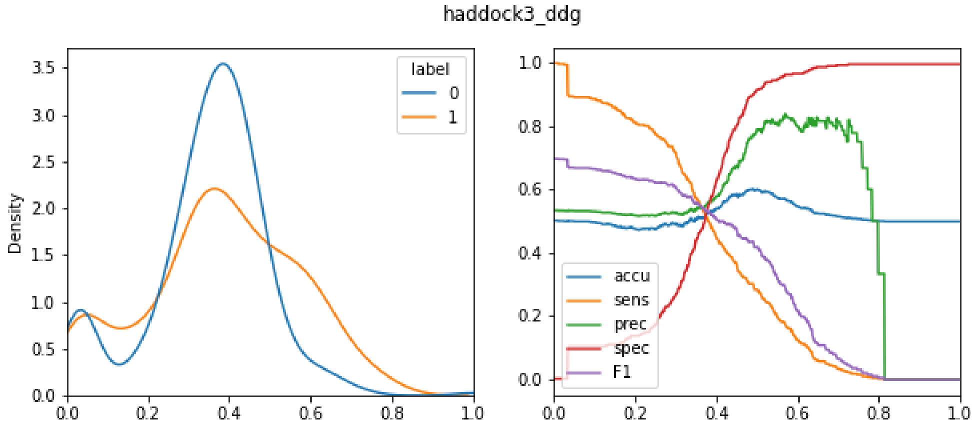
haddock3_ddg
Density
label
0
1
accu
sens
prec
spec
F1

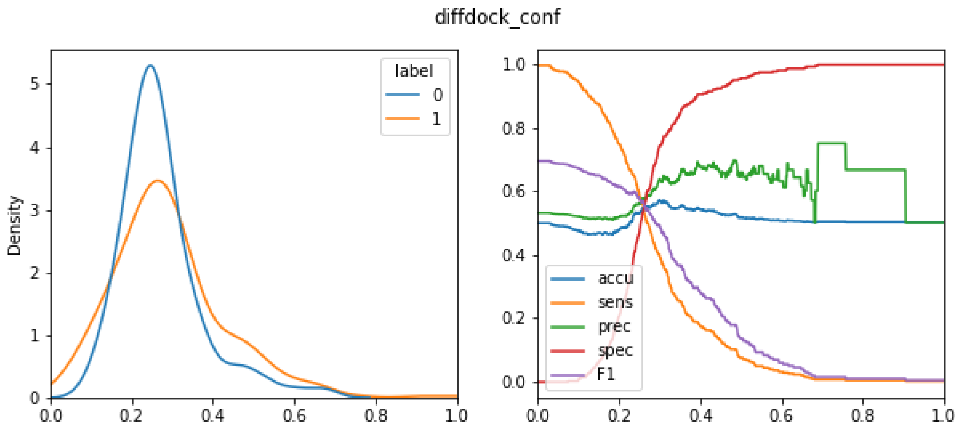


*Scores from each method were normalised between 0 and 1, corresponding to the lowest and highest scoring samples. Distributions of normalised score plot as kernel density estimate. Positive sample shows in orange and negative sample shows in blue. (right) Relationship between normalized threshold value and model indicators. accu: accuracy, sens: sensitivity, spec: specificity, prec: precision, F1: F1 score. pred_score: CLIMBS score, diffdock_conf: DIFFDOCK confidence, Rosetta_ddg: Rosetta binding free energy, autodock4_ddg: Autodock4 binding free energy, haddock3_ddg: HADDOCK3 binding free energy.*

## *Supplementary Data 9. Details of new sugar retraining*

**A)**

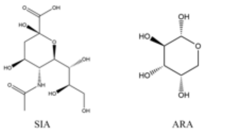


| | SIA (37) | | ARA (23) | |
|---|---|---|---|---|
| | accuracy | loss | accuracy | loss |
| initial database | 67.57% | 0.318 | 78.26% | 0.21 |
| +12 SIA sample | 81.08% | 0.226 | 95.65% | 0.088 |
| +24 SIA sample | 78.38% | 0.248 | 78.26% | 0.232 |
| +48 SIA sample | **91.89%** | 0.092 | 73.91% | 0.251 |

**B)**

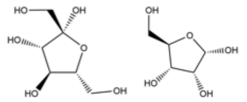


| | FRU (17) | | RIB (17) | |
|---|---|---|---|---|
| | accuracy | loss | accuracy | loss |
| initial database | 56.52% | 0.426 | 88.24% | 0.108 |
| +12 FRU sample | 60.87% | 0.414 | 94.12% | 0.195 |
| +24 FRU sample | 56.52% | 0.429 | 88.24% | 0.136 |
| +48 FRU sample | **95.65%** | 0.048 | 82.35% | 0.198 |

**C)**

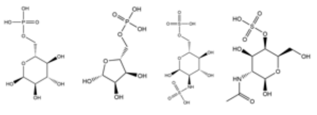


| | G6P (14) | | RP5 (21) | |
|---|---|---|---|---|
| | accuracy | loss | accuracy | loss |
| initial database | 71.43% | 0.27 | 76.19% | 0.24 |
| +12 G6P sample | **78.57%** | 0.23 | 85.71% | 0.15 |

| | SGN (33) | | ASG (39) | |
|---|---|---|---|---|
| | accuracy | loss | accuracy | loss |
| initial database | 60.61% | 0.39 | 74.36% | 0.26 |
| +37 SGN sample | **81.82%** | 0.19 | 76.92% | 0.23 |

**D)**

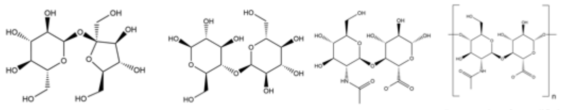


| | Sucrose (58) | | Maltose (53) | | GlcNAc-GlcA (78) | | unmodified HS (8) | |
|---|---|---|---|---|---|---|---|---|
| | accuracy | loss | accuracy | loss | accuracy | loss | accuracy | loss |
| initial database | 68.97% | 0.31 | 66.04% | 0.34 | 91.03% | 0.09 | 75.00% | 0.22 |
| +12 di sample | 72.41% | 0.27 | 73.58% | 0.26 | 89.74% | 0.16 | 87.50% | 0.11 |
| +24 di sample | 82.76% | 0.21 | 73.58% | 0.28 | 87.18% | 0.16 | 87.50% | 0.11 |
| +48 di sample | 82.76% | 0.2 | **77.36%** | 0.24 | 80.77% | 0.21 | 87.50% | 0.1 |
| +96 di sample | **87.93%** | 0.12 | 66.04% | 0.33 | 91.03% | 0.16 | 87.50% | 0.11 |

*Supplementary figure 1 Retraining CLIMBS with new sugars. A. Accuracy and training loss of arabinose (ARA, 23 samples in test set) and sialic acid samples (SIA, 37 samples in test set) from a CLIMBS model trained on the initial database plus a different number of sialic acid samples (see Supplementary Data 3f). B. Accuracy and training loss of ribose (RIB, 17 samples in test set) and fructose (FRU, 17 samples in test set) samples from a CLIMBS model trained*

*on the initial database plus different numbers of fructose samples (see Supplementary Data 3g). C. (Above) Accuracy and training loss of 6-O-phosphono-glucose (G6P, 14 samples in test set), 5-O-phosphono-ribose (RP5, 21 samples in test set) from a CLIMBS model trained on the initial database plus a different number of 6-O-phosphono-glucose samples. (Bottom) Accuracy and training loss of N,O6-disulfo-glucosamine (SGN, 33 samples in test set), N-acetyl-4-O-sulfo-galactosamine (ASG, 39 samples in test set) and trained. D. Accuracy and training loss of maltose, sucrose, GlcNAc-GlcA, and repeat regions of unmodified heparan sulphate samples from a CLIMBS model trained on the initial database plus different numbers of maltose and sucrose samples (See Supplementary Data 3i). The ratio of maltose and sucrose is 1:1. HS: heparan sulphate.*

*Supplementary Data 10. CLIMBS on docking and design problems*

The carbohydrate-protein complexes were generated by Chai-1 (*12*). Protein amino acid sequence and carbohydrate SMILES sequence were input without MSA, template, and restraints. For docking, the rank 0 model of each carbohydrate-protein complex with protein pLDDT>90, ligand pLDDT>70, protein backbone RMSD <2 Å to the reference structure was kept. For design, the rank 0 model of each carbohydrate-protein complex with protein pLDDT>90, ligand pLDDT>70 was kept. Evaluation by metrics was performed on the Rosetta relaxed structure.

CLIMBS performance was tested on the *db_dock* dataset and compared with Rosetta ddg at different energy cutoffs. CLIMBS_D was a model trained on a selected number of complexes with resolution < 2 Å (*db_w2*).

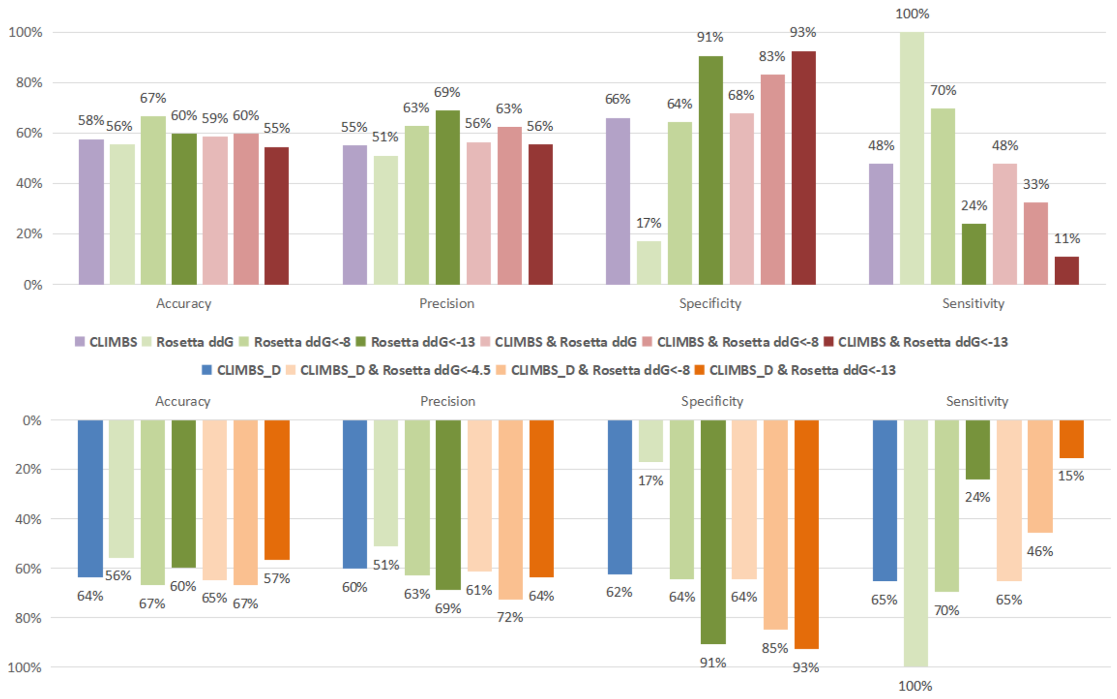


*Supplementary Figure 7 Performance of CLIMBS, CLIMBS_D, and Rosetta on the carbohydrate-protein docking problem. CLIMBS_D was trained with fewer samples with high structure quality (db_w2, see Supplementary Data 31). 99 samples were included. Structures with ligand RMSD <2 Å after superimposing the protein to the reference structure are regarded as bound. Thresholds to predict as positive for each metric: CLIMBS=1, CLIMBS_D=1, Rosetta score function ddG <-4.5, <-8, and <-13. The positive predictions of combined metrics are the AND subset of each positive prediction. accuracy: (TP+TN)/(TP+FP+TN+FN), precision: TP/(TP+FP), specificity: TN/(TN+FP), sensitivity: TP/(TP+FN). TP: True Positive, TN: True Negative, FP: False Positive, FN: False Negative.*

*Supplementary Table 5 Label of carbohydrate-protein complexes in the design problem.1: bound, 0: unbound. Previous research (13) did a cross-binding assay between different carbohydrates and 4 protein variants. Carbohydrate-protein results with y-median fluorescence intensity higher than 5000 were regarded as bound.*

| | 08F | 13D | 24i | 83H |
|---|---|---|---|---|
| Chitobiose | 1 | 1 | 1 | 0 |
| Core5 | 0 | 1 | 1 | 0 |
| Core8 | 0 | 0 | 1 | 0 |
| GAL | 0 | 0 | 0 | 0 |
| GalNAc | 0 | 0 | 1 | 0 |
| GlcNAc | 0 | 0 | 0 | 0 |
| H3 | 0 | 0 | 1 | 0 |
| LacNAc | 0 | 0 | 0 | 0 |
| Lec | 1 | 1 | 1 | 0 |
| Man | 0 | 0 | 0 | 0 |
| Neu5Ac | 0 | 0 | 0 | 0 |
| Neu5Gc | 0 | 0 | 0 | 0 |
| TF | 1 | 1 | 0 | 0 |

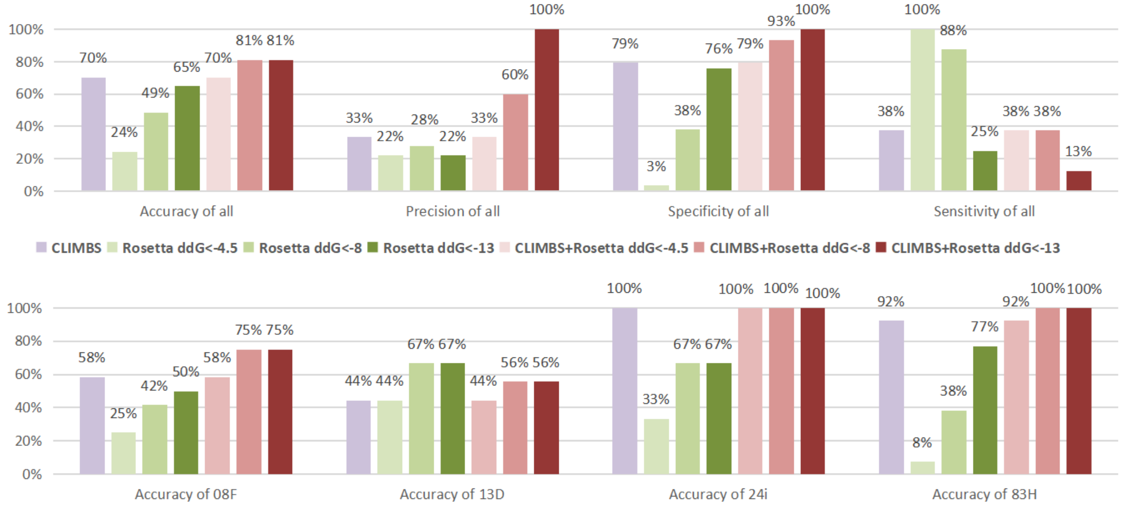


*Supplementary Figure 8 Performance of different scoring methods on the design carbohydrate-binding protein problem. In total, 37 samples were included: 12 samples of 08F, 9 samples of 13D, 3 samples of 24i, and 13 samples of 83H. Four protein variants bind to different carbohydrates in 13 candidates. Thresholds to predict as positive for each metric: CLIMBS=1, Rosetta score function ddG <-4.5, <-8, and <-13. The positive predictions of combined metrics are the AND subset of each positive prediction. accuracy: (TP+TN)/(TP+FP+TN+FN), precision: TP/(TP+FP), specificity: TN/(TN+FP), sensitivity: TP/(TP+FN). TP: True Positive, TN: True Negative, FP: False Positive, FN: False Negative*

*Supplementary Data 11. Counterfactual test*

Counterfactual experiment was done on a subset from positive test set. Initially, 100 beta-glucose-included complexes were selected, a series of stereochemistry perturbations were introduced to the glucose. Then, the complexes went a short Rosetta relaxation that fixes protein structures in order to refine the local structure after change. To select the complexes that perturbations and relaxations did not introduce substantial changes to the position or orientation of the sugars, relaxed complexes were filtered by requiring that the sugar backbone RMSD between the structures before and after relaxation be < 0.5 Å, and 68 complexes were kept. Those complexes were then evaluated by CLIMBS.

There are a few reasons to choose complexes with beta-glucoses for counterfactual experiment. The first reason is that glucose is the most common sugar in life, participating in various biological process. The second is because the complexes with beta-glucose is the largest class in the database. Thirdly, the beta-glucose is the most standard monosaccharide that all hydrogen groups on sugar backbone are oriented on equatorial position.

*Supplementary Figure 9: Counterfactual perturbations applied to β-glucose in protein–carbohydrate complexes.*

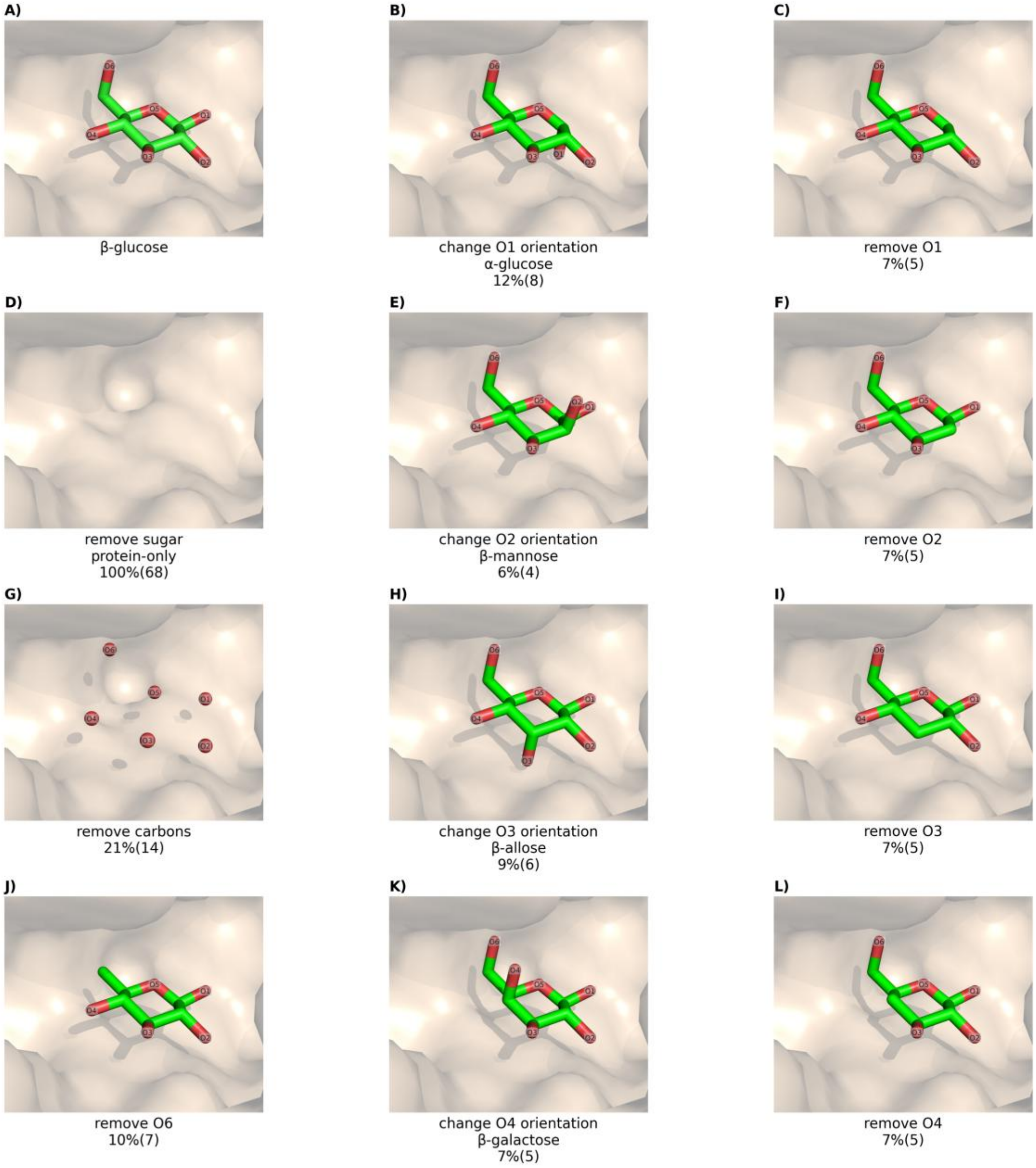


*Representative counterfactual manipulations performed on β-glucose within the binding pocket are illustrated. (A) Native β-glucose bound in the protein cavity is shown as the reference configuration. (B–C) Perturbations at the anomeric position include flipping the O1 hydroxyl orientation to generate the α-glucose configuration and removing the O1 hydroxyl group. (D) Complete removal of the sugar ligand (protein-only control). (E–F) Modifications at O2 include inversion of the hydroxyl orientation corresponding to the β-mannose stereochemistry and removal of the O2 hydroxyl group. (G) Removal of all carbon atoms of the sugar ring while retaining the surrounding environment, eliminating potential CH–π interactions. (H–I) Perturbations at O3 include hydroxyl orientation inversion corresponding to β-allose and removal of the O3 hydroxyl group. (J) Removal of the terminal O6 hydroxymethyl group. (K–L) Modifications at O4 include orientation inversion corresponding to β-galactose and removal of the O4 hydroxyl group. The percentages shown below each panel indicate the fraction of complexes whose predicted binding classification changed after the perturbation. 68 samples were included.*

*Supplementary Data 12. Graph statistics and shortcut signal tests*

Graph statistics and shortcut signal tests were performed using the entire *db_w1* dataset (Supplementary Data 3a), consisting of 2838 positive and 2530 negative samples. Eight structural features were analysed: six features included in the input graph (number of atoms, fragments, molecules, chemical bonds, polar interaction, and CH–π interactions) and two features excluded during preprocessing (the number of residues within 4 Å of the sugar and residues within 4 Å that were not captured in the graph).

Graph statistics (Supplementary Figure 10) show that, compared with negative samples, positive samples tend to contain more interacting residues (polar and CH–π interactions) but generally fewer total residues surrounding the sugar. Importantly, these surrounding residues are excluded during preprocessing. As a result, the preprocessing step reduces the overall graph size while focusing the representation on functionally relevant nodes and edges. This design is consistent with the original goal of incorporating biochemical and structural knowledge to guide and facilitate model training.

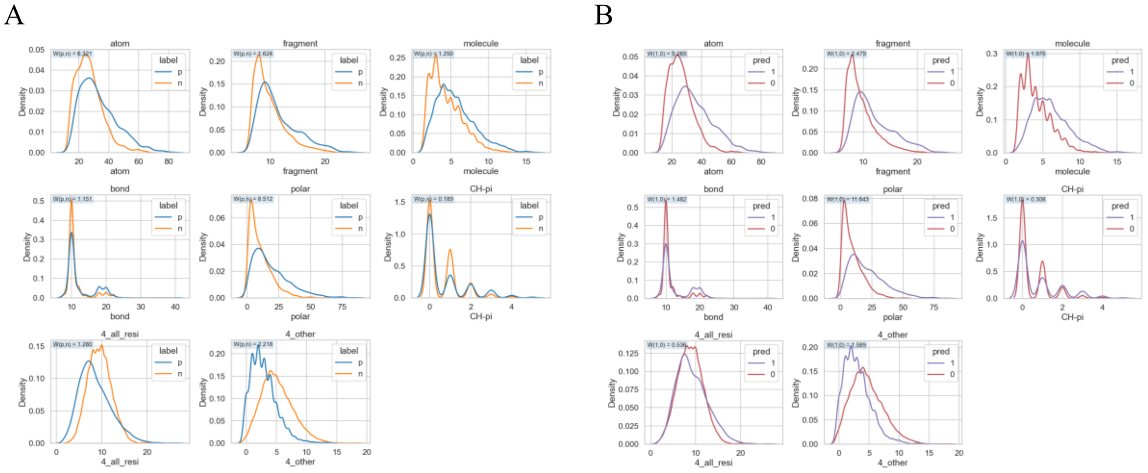


*Supplementary Figure 10: Graph statistics of CLIMBS inputs classified by positive and negative labels (A), and binding and non-binding prediction by CLIMBS (B). atom: atom count in graph ; fragment: fragment count in graph ; molecule: residue count in graph ; bond: chemical bond count in graph ; polar: polar interaction count in graph ; CH-pi: CH-π interaction count in graph ; 4_all_resi: count of all residues within 4 Å of the sugar, including residues not captured in graph ; 4_other: count of residues within 4 Å that were not captured in the graph. 5368 samples were*

*included.*

To further evaluate whether preprocessing introduces graph-size shortcut signals, a shortcut signal test using simple baseline models based solely on the counts of eight graph-related features was conducted. As shown in Supplementary Figure 11, the strongest baseline signal comes from the number of atoms (AUROC = 0.651, AUPRC = 0.686), while other features such as the number of uncaptured residues within 4 Å show even weaker predictive ability (AUROC = 0.248, AUPRC = 0.407). However, the performance of all such baselines remains substantially lower than that of CLIMBS. These results indicate that, although positive samples may produce slightly larger graphs after preprocessing, the predictive performance of CLIMBS cannot be explained by simple graph-size statistics. Instead, the model learns more informative structural patterns associated with carbohydrate–protein interactions.

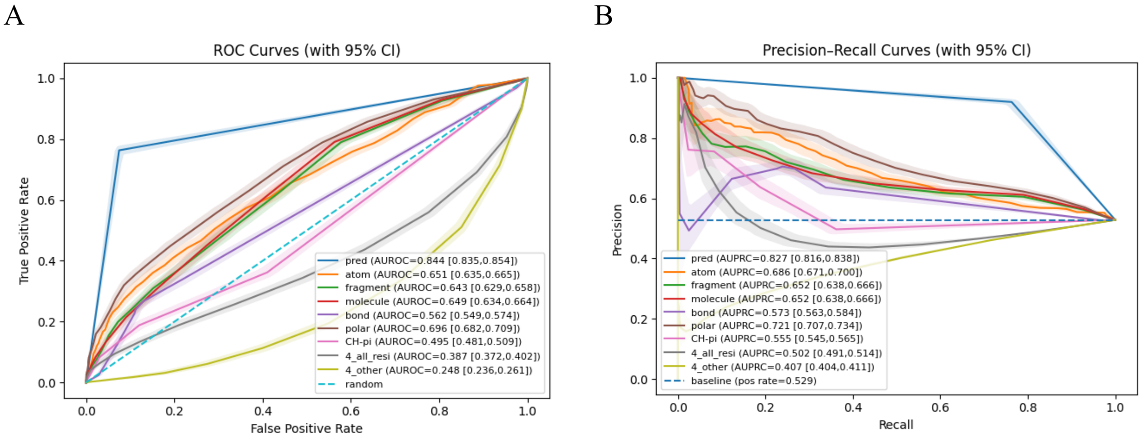


*Supplementary Figure 11: (A) ROC curves and (B) PR curves of Shortcut signal test with 95% confidence intervals. pred: CLIMBS prediction; atom: atom count in graph ; fragment: fragment count in graph ; molecule: residue count in graph ; bond: chemical bond count in graph ; polar: polar interaction count in graph ; CH-pi: CH-π interaction count in graph ; 4_all_resi: count of all residues within 4 Å of the sugar, including residues not captured in graph ; 4_other: count of residues within 4 Å that were not captured in the graph. 5368 samples were included.*

*Supplementary method 1. Rosetta relaxation and docking(14)*

a. Rosetta relaxation command

```
rosetta_scripts.linuxgccrelease -nstruct 1 -parser:protocol single_sugar_relax_fast.xml -
include_current -ignore_unrecognized_res -include_sugars -s file_name
```

*b.* Rosetta relaxation *script single_sugar_relax_fast.xml*

```
<ROSETTASCRIPTS>
        <RESIDUE_SELECTORS>
    <Chain name='sugar' chains='A' />
    <Not name="protein" selector="sugar"/>
    <InterfaceByVector name="strict_interface" grp1_selector="sugar"
grp2_selector="protein"/>
    <PrimarySequenceNeighborhood name="interface" selector="strict_interface"/>
   </RESIDUE_SELECTORS>
        <MOVERS>
      <EnsureExclusivelySharedJumpMover name="set_sugar_other_jump"
residue_selector="sugar" />
      <AtomTree name="set_foldtree"   docking_ft="1" />
      <InterfaceAnalyzerMover name='interface_analyis' ligandchain='A'
interface_sc='1' tracer='0'/>
         </MOVERS>
         <FILTERS>
```

```
<Ddg name="ddg" confidence="0" repeats='1' />
</FILTERS>
<PROTOCOLS>
<Add mover_name="set_foldtree"/>
<Add mover_name="set_sugar_other_jump"/>
<Add mover="relax"/>
<Add mover="interface_analyis" />
<Add filter="ddg"/>
</PROTOCOLS>
<OUTPUT />
</ROSETTASCRIPTS>
```

c. Rosetta docking command

```
rosetta_scripts.linuxgccrelease -s file_name -in:file:native file_name -parser:protocol local_ligand_dock.xml -nstruct 200 -include_sugars -ex1 -ex2
```

d. Rosetta docking script *local_ligand_dock.xml*

```
<ROSETTASCRIPTS>
  <SCOREFXNS>
    <ScoreFunction name="ligand_soft_rep" weights="ligand_soft_rep">
      <Reweight scoretype="fa_elec" weight="0.42"/>
      <Reweight scoretype="hbond_bb_sc" weight="1.3"/>
      <Reweight scoretype="hbond_sc" weight="1.3"/>
```

```
        <Reweight scoretype="rama" weight="0.2"/>
      </ScoreFunction >
      <ScoreFunction name="hard_rep" weights="ligand">
        <Reweight scoretype="fa_intra_rep" weight="0.004"/>
        <Reweight scoretype="fa_elec" weight="0.42"/>
        <Reweight scoretype="hbond_bb_sc" weight="1.3"/>
        <Reweight scoretype="hbond_sc" weight="1.3"/>
        <Reweight scoretype="rama" weight="0.2"/>
      </ScoreFunction>
    </SCOREFXNS>
    <LIGAND_AREAS>
      <LigandArea name="docking_sidechain" chain="X" cutoff="6.0"
add_nbr_radius="true" all_atom_mode="true" minimize_ligand="10"/>
      <LigandArea name="final_sidechain" chain="X" cutoff="6.0"
add_nbr_radius="true" all_atom_mode="true"/>
      <LigandArea name="final_backbone" chain="X" cutoff="7.0"
add_nbr_radius="false" all_atom_mode="true" Calpha_restraints="0.3"/>
    </LIGAND_AREAS>
    <INTERFACE_BUILDERS>
      <InterfaceBuilder name="side_chain_for_docking"
ligand_areas="docking_sidechain"/>
      <InterfaceBuilder name="side_chain_for_final"
ligand_areas="final_sidechain"/>
```

```
        <InterfaceBuilder name="backbone" ligand_areas="final_backbone"
extension_window="3"/>
    </INTERFACE_BUILDERS>
    <MOVEMAP_BUILDERS>
        <MoveMapBuilder  name="docking"
sc_interface="side_chain_for_docking" minimize_water="true"/>
        <MoveMapBuilder  name="final" sc_interface="side_chain_for_final"
bb_interface="backbone" minimize_water="true"/>
    </MOVEMAP_BUILDERS>
    <RESIDUE_SELECTORS>
        <InterfaceByVector name="interface">
         <Chain chains="X"/>
         <Chain chains="B"/>
        </InterfaceByVector>
    </RESIDUE_SELECTORS>
    <FILTERS>
        <Ddg name="ddg" confidence="0"/>
        <Sasa name="SASA" confidence="0"/>
        <ResidueCount name="resi_interface" residue_selector="interface"
confidence="0"/>
    </FILTERS>
    <MOVERS>
        <Translate name="translate" chain="X" distribution="uniform"
```

```
angstroms="5.0" cycles="50" force="true"/>
    <Rotate name="rotate" chain="X" distribution="uniform" degrees="360"
cycles="500"/>
    <SlideTogether name="slide_together" chains="X"/>
    <HighResDocker name="high_res_docker" cycles="1"
repack_every_Nth="1" scorefxn="ligand_soft_rep"
movemap_builder="docking"/>
    <FinalMinimizer name="final" scorefxn="hard_rep"
movemap_builder="final"/>
    <InterfaceScoreCalculator name="add_scores" chains="X"
scorefxn="hard_rep"/>
    <AddJobPairData name="system_name" key="system_name"
value_type="string" value_from_ligand_chain="X" />
    <ParsedProtocol name="low_res_dock">
      <Add mover_name="translate"/>
      <Add mover_name="rotate"/>
      <Add mover_name="slide_together"/>
    </ParsedProtocol>
    <ParsedProtocol name="high_res_dock">
      <Add mover_name="high_res_docker"/>
      <Add mover_name="final"/>
    </ParsedProtocol>
    <ParsedProtocol name="reporting">
```

```
        <Add mover_name="add_scores"/>
      </ParsedProtocol>
  </MOVERS>
  <PROTOCOLS>
    <Add mover_name="high_res_dock"/>
     <Add mover_name="reporting"/>
     <Add filter="ddg" />
     <Add filter="SASA" />
     <Add filter="resi_interface" />
  </PROTOCOLS>
</ROSETTASCRIPTS>
```

*References*


1. K. L. Hudson *et al.*, Carbohydrate-Aromatic Interactions in Proteins. *J Am Chem Soc* **137**, 15152-15160 (2015).
2. A. Leaver-Fay *et al.*, in *Methods in Enzymology,* M. L. Johnson, L. Brand, Eds. (Academic Press, 2011), vol. 487, pp. 545-574.
3. H. M. Berman *et al.*, The Protein Data Bank. *Nucleic Acids Research* **28**, 235-242 (2000).
4. F. P. Yijie Luo, Carbohydrate-protein complexes library. *Zenodo*, (2025).
5. R. J. Read *et al.*, A new generation of crystallographic validation tools for the protein data bank. *Structure* **19**, 1395-1412 (2011).
6. O. S. Smart *et al.*, Validation of ligands in macromolecular structures determined by X-ray crystallography. *Acta Crystallographica Section D* **74**, 228-236 (2018).
7. S. Guo *et al.*, Structural Basis of Ligand Selectivity by a Bacterial Adhesin Lectin Involved in Multispecies Biofilm Formation. *mBio* **12**, 10.1128/mbio.00130-00121 (2021).
8. R. F. Alford *et al.*, The Rosetta All-Atom Energy Function for Macromolecular Modeling and Design. *J Chem Theory Comput* **13**, 3031-3048 (2017).

9. G. M. Morris *et al.*, AutoDock4 and AutoDockTools4: Automated docking with selective receptor flexibility. *J Comput Chem* **30**, 2785-2791 (2009).
10. C. Dominguez, R. Boelens, A. M. J. J. Bonvin, HADDOCK:  A Protein−Protein Docking Approach Based on Biochemical or Biophysical Information. *Journal of the American Chemical Society* **125**, 1731-1737 (2003).
11. G. a. S. Corso, Hannes and Jing, Bowen and Barzilay, Regina and Jaakkola, Tommi, DiffDock: Diffusion Steps, Twists, and Turns for Molecular Docking. *International Conference on Learning Representations (ICLR)*,  (2023).
12. C. D. team *et al.*, Chai-1: Decoding the molecular interactions of life. *bioRxiv*, 2024.2010.2010.615955 (2024).
13. E. M. Ward *et al.*, Engineered Glycan-Binding Proteins for Recognition of the Thomsen-Friedenreich Antigen and Structurally Related Disaccharides. *ACS Chem Biol* **18**, 70-80 (2023).
14. S. J. Fleishman *et al.*, RosettaScripts: a scripting language interface to the Rosetta macromolecular modeling suite. *PLoS One* **6**, e20161 (2011).
